# New functions of Semaphorin 3E and its receptor PlexinD1 during developing and adult hippocampal formation

**DOI:** 10.1038/s41598-018-19794-0

**Published:** 2018-01-22

**Authors:** Agata Mata, Vanessa Gil, Jeús Pérez-Clausell, Miguel Dasilva, Mari Carmen González-Calixto, Eduardo Soriano, José Manuel García-Verdugo, Maria V. Sanchez-Vives, José Antonio del Río

**Affiliations:** 1 Molecular and Cellular Neurobiotechnology, Institute for Bioengineering of Catalonia (IBEC), The Barcelona Institute of Science and Technology, Parc Científic de Barcelona, Barcelona, Spain; 2 Department of Cell Biology, Physiology and Immunology, Universitat de Barcelona, Barcelona, Spain; 3 Centro de Investigación Biomédica en Red sobre Enfermedades Neurodegenerativas, (CIBERNED), Barcelona, Spain; 4 Institut de Neurociències de la Universitat de Barcelona, Barcelona, Spain; 5 Systems Neuroscience, Institut d’Investigacions Biomèdiques August Pi i Sunyer (IDIBAPS), Barcelona, Spain; 6 ICREA, Barcelona, Spain; 7 Laboratory of Comparative Neurobiology, Institute Cavanilles, University of Valencia, CIBERNED, 46980 Valencia, Spain; 8 Vall d’Hebrón Institut de Recerca (VHIR), Barcelona, Spain

## Abstract

The development and maturation of cortical circuits relies on the coordinated actions of long and short range axonal guidance cues. In this regard, the class 3 semaphorins and their receptors have been seen to be involved in the development and maturation of the hippocampal connections. However, although the role of most of their family members have been described, very few data about the participation of Semaphorin 3E (Sema3E) and its receptor PlexinD1 during the development and maturation of the entorhino-hippocampal (EH) connection are available. In the present study, we focused on determining their roles both during development and in adulthood. We determined a relevant role for Sema3E/PlexinD1 in the layer-specific development of the EH connection. Indeed, mice lacking Sema3E/PlexinD1 signalling showed aberrant layering of entorhinal axons in the hippocampus during embryonic and perinatal stages. In addition, absence of Sema3E/PlexinD1 signalling results in further changes in postnatal and adult hippocampal formation, such as numerous misrouted ectopic mossy fibers. More relevantly, we describe how subgranular cells express PlexinD1 and how the absence of Sema3E induces a dysregulation of the proliferation of dentate gyrus progenitors leading to the presence of ectopic cells in the molecular layer. Lastly, Sema3E mutant mice displayed increased network excitability both in the dentate gyrus and the hippocampus proper.

## Introduction

The hippocampal formation plays crucial roles in the consolidation of information from short- to long-term memory, as well as in spatial memory^1,2^. Since different neuronal cell types, the main extrinsic afferent connections (i.e., entorhinal or commissural/associational fibers) and the most relevant intrinsic connection (i.e., the mossy fibers) are organized into well-defined lamina in the hippocampus^3^, it has frequently been used as a system model for studying fundamental neuroscience including neurophysiology^4^. During axonal wiring in perinatal development in rodents, entorhinal axons refrain from invading the adjacent ventrolateral isocortex, entering the hippocampal formation to reach the *stratum lacunosum moleculare* (slm) of the hippocampus proper to further innervate the outermost portion of the molecular layer (oml) of the *fascia dentata*^5–8^. In addition, axons from isocortical regions close to the entorhinal cortex (ventrolateral neocortex) avoid the hippocampal region (including hippocampal and retrohippocampal formation) (e.g.^7^). In fact, based on classical neuroanatomical studies using axonal tracers (e.g.^5–7^), cell transplantation *in vivo* (e.g.^9–11^) and *in vitro* in slices (e.g.^12–17^), it has been suggested that axons from entorhinal neurons are able to reach specifically the slm/oml of the hippocampal formation in healthy conditions. In fact, commissural/associational axons avoid the slm/oml *in vivo*^5–7,18^, as well as *in vitro*^16,19,20^, being restricted to the *stratum radiatum* of the hippocampus proper and the innermost portion of the molecular layer (iml) of the dentate gyrus^8^. With respect to mossy fibers, postnatal and adult newborn granule cells extend their axons, forming synaptic contacts on hilar mossy cells and on proximal dendrites of the CA3 pyramidal cells in the *stratum lucidum*^3,8^.

Axonal specification at the hippocampus is a highly orchestrated process regulated by a large number of factors, both at the topographic and local levels, that have been sequentially revealed for years (e.g.^21–23^). One of these molecule families, semaphorins and their receptors neuropilins as well as their co-receptors, plexins, have emerged as important cellular cues regulating key developmental processes, such as neuronal migration and axonal guidance^24–26^. In particular, during hippocampal development, the participation of class III semaphorins in the ingrowth and maturation of entorhino-hippocampal (EH) (mainly Sema3A and 3F), septo-hippocampal (Sema3C) or subicular connections (Sema3E) has been described^27–32^. In addition, Sema3F together with its receptor complex Neuropilin2/PlexinA3 actively participate in the maturation of the hippocampal mossy fibers^33–35^. However, the participation of Sema3E and its receptor PlexinD1 in the development of the hippocampal connections has not yet been analysed in detail, and differing data have been published. Indeed, Pozas *et al*., showed that in mice Sema3E repels exclusively hippocampal axons but only at embryonic day 14.5 (E14.5) without effects from E14.5 onwards^30^. This was not observed by Chauvet *et al*., and Deck *et al*., who found that Sema3E is able to collapse cortical axons around E17.5^32^ or E14.5-16.5^36^, respectively.

Functions of Sema3E/PlexinD1 are also associated with vascular development and remodelling, and axon regeneration after injury or cancer (e.g.^37–42^). Indeed, as described for other signalling mechanisms (i.e., Netrins^43^), Sema3E/PlexinD1 might play several roles in different cell types during development and in the adult. Keeping this in mind, in the present study we analyse in detail the participation of Sema3E and its receptor PlexinD1 during development and in adult hippocampal region. We focus our experiments on analysis of the afferent connection (EH-connection) during development as well as on the correlation of the putative changes with the laminar distribution of hippocampal afferents in the adult. Moreover, we explore cell-specific functions of Sema3E/PlexinD1 in the hippocampal formation. Considering that semaphorins have been described as being involved in cell proliferation (e.g.^44,45^), we focus our attention on analysing whether absence of Sema3E-mediated signalling modifies cell proliferation in the adult hippocampus and putative effects on mossy fiber growth and hippocampal physiology. Our results indicate that Sema3E and PlexinD1 participate actively in the establishment of the EH connection by regulating the laminar termination of ingrowing entorhinal axons in the hippocampus. In addition, the absence of Sema3E/PlexinD1 signalling in postnatal and adult stages also lead to changes in the cytoarchitecture of the dentate gyrus. In their absence, an increased number of stem cell niches are observed in the subgranular zone and newborn granule cells are ectopically settled in the molecular layer. This disorganization strongly alters the laminar distribution and synaptic patterning of mossy fibers. Lastly, these modifications also correlate with the presence of altered functional properties as measured by means of multielectrode local field potential (LFP) recordings, revealing an enhanced excitability in *Sema3E*^*0/0*^ mice.

## Results

### Expression pattern of secreted semaphorins and their receptors in the developing hippocampal formation

**Figure 1:**
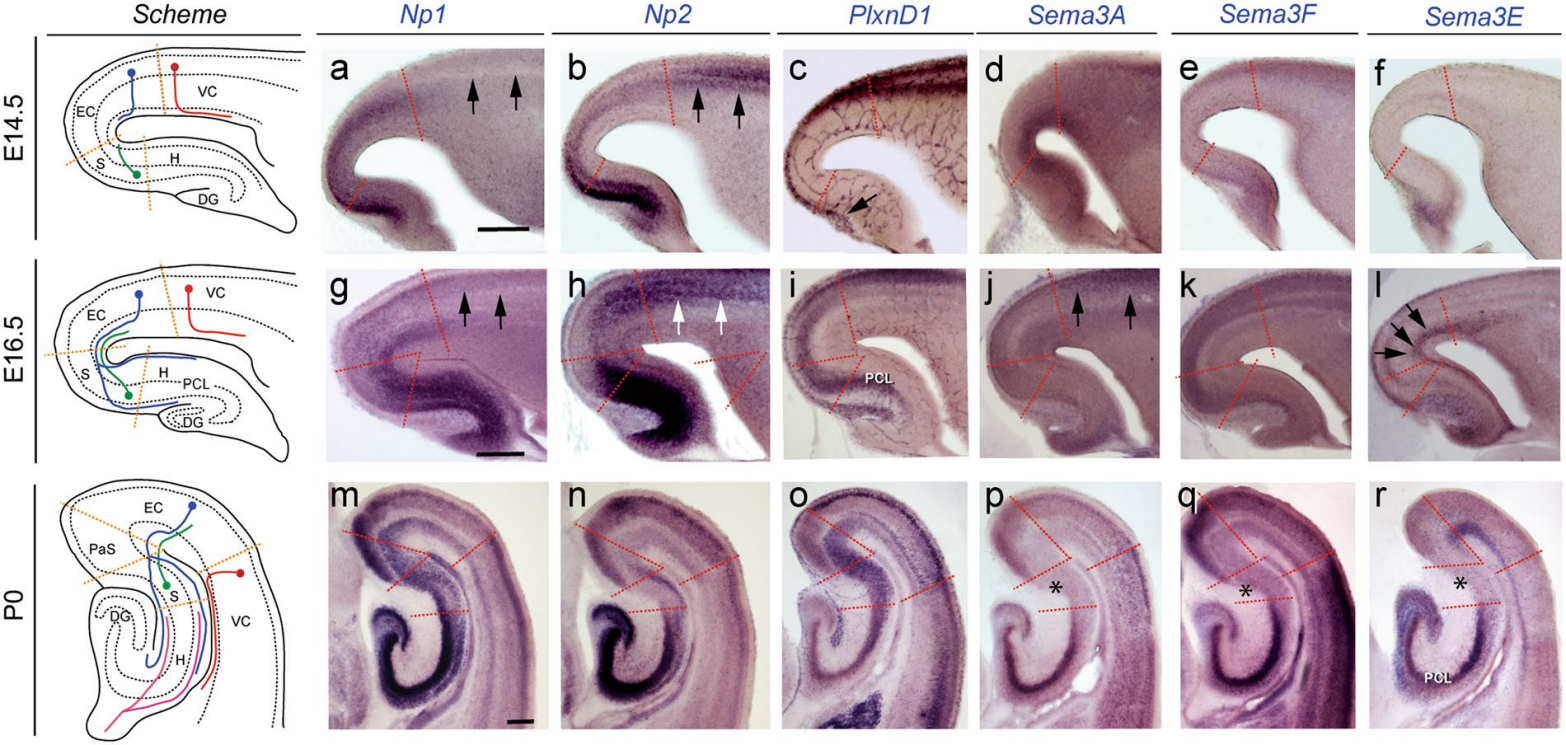
Low-power photomicrographs illustrating the distribution of *Np1* (**a,g,m**); *Np2* (**b,h,n**); *PlnxD1* (**c,i,o**); *Sema3A* (**d,j,p**); *Sema3F* (**e,k,q**) and *Sema3E* (**f,l,r**) mRNA in the hippocampal formation and adjacent ventrolateral cortex at E14.5 (**a–f**), E16.5 (**g–l**) and P0 (**m–r**). The different regional boundaries are circumscribed by dashed lines. Characteristic corticofugal, entorhino-hippocampal, subiculo-entorhinal and commissural afferent connections are labelled in red, blue, green and pink respectively in the scheme. Note the absence of *Np1* labelling in the ventrolateral cortex at E14.5 and E16.5 (arrows in **a** and **g**), compared to *Np2* (arrows in **b** and **h**) and *PlnxD1* (**c** and **i**). *Sema3A* levels in the ventrolateral neocortex were intense at E16.5 (arrows in **j**). In addition, *Sema3E* levels in lower layers of both the ventrolateral and entorhinal cortices can be seen from E16.5 onwards (arrows in **l**). Surprisingly, the subicular region was almost absent of semaphorin labelling (asterisk in **p**–**r**). Abbreviations: DG = dentate gyrus; EC = entorhinal cortex; H = hippocampus proper; PaS = parasubiculum; PCL = pyramidal cell layer; S = subiculum; VC = ventrolateral neocortex. Scale bars: **a** = 250 μm pertains to (**b**–**f**); **g** = 250 μm pertains to (**h**–**l**) and **m** = 100 μm pertains to (**n**–**r**).

Figure 1 shows the low-magnification views of *Sema3A*, *Sema3F*, *Sema3E*, *Np1*, *Np2* and *PlxnD1* mRNAs expression in the developing hippocampal formation of E14.5, E16.5 and P0 mice. Due to the anatomical distribution of entorhinal fibers entering the hippocampus^5–8^, we histologically processed horizontal brain sections of the hippocampal formation for *in situ* hybridization (Fig. 1). Detailed analyses of *Sema3A*, *Sema3F*, *Np1* and *Np2* distribution showed it was similar to that previously reported^28^ although with some differences. Developed sections indicated that both *Np1* and *Np2* were strongly expressed in the hippocampus between E14.5-E16.5 with decreased levels in the adjacent entorhinal cortex. *Np1* labelling, in contrast to *Np2*, was intense in the entorhinal cortex at E14.5, and absent in the adjacent ventrolateral neocortex between E14.5 and E16.5. In addition, *Np1* mRNA labelling was intense in the subiculum compared to *Np2* at P0. For ligands, *Sema3A* staining was relevant in the adjacent ventrolateral neocortex at E14.5-P0, and *Sema3F* levels in the entorhinal cortex increased from E14.5 to P0. In the hippocampus, Sema3A and 3F displayed similar patterns of labelling from E14.5 to P0 with the pyramidal layer of CA1-3 intensely labelled. Relevantly, the subicular region showed the lowest staining of all three semaphorins in the hippocampal formation at P0 (asterisk in Fig. 1p–r).

*PlxnD1* labelling appeared in hippocampal Cajal-Retzius cells at E14.5 (arrow in Fig. 1c), and from E16.5 onwards in the pyramidal cell layer (PCL) of the subiculum-CA1, as previously described^32,41^. In contrast, *Sema3E* transcript was lightly detected in the emerging dentate gyrus between E14.5-E16.5 and from P0 onwards in the PCL of CA1. In ventrolateral neocortex and entorhinal cortex, *PlxnD1* mRNA appeared in upper cortical layers following a lateral to medial gradient of intensity at E14.5. In contrast, *Sema3E* was present in lower layers of the entorhinal cortex close to the ventricular region from E16.5 (arrows in Fig. 1l) to P0. Sense controls displayed no specific signals (Supplementary Fig. 1). In summary, these results indicate that, as reported by Sema3A/3 F and Np1/2^21,28^, PlexinD1 and Sema3E are expressed in the hippocampal formation during the development of the entorhino-hippocampal connection.

### Chemorepulsive action of Sema3E on entorhinal and hippocampal axons during embryonic development

**Figure 2:**
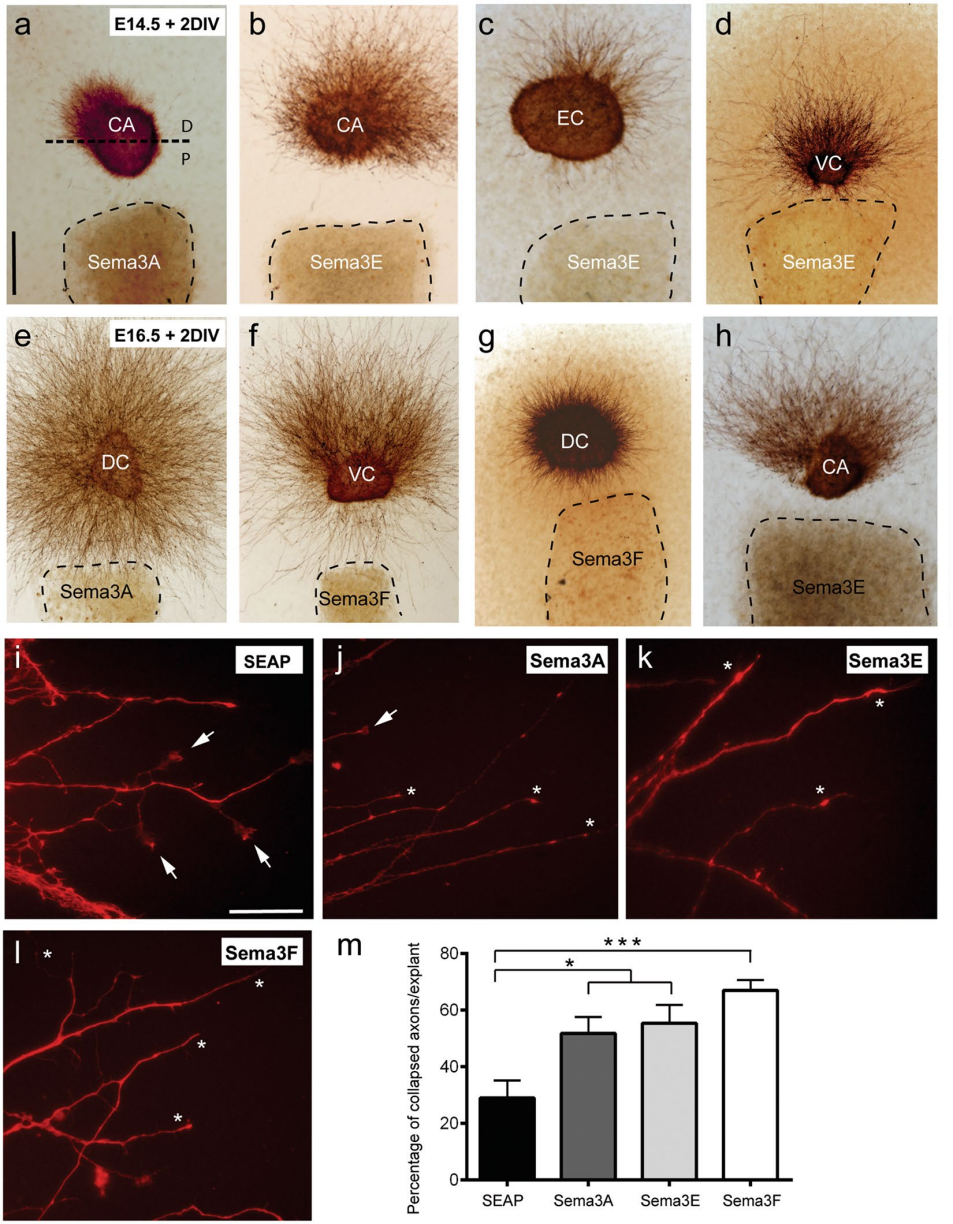
Low-power photomicrographs showing examples of the chemorepulsion of hippocampal (**a,b**,**h**), entorhinal (**c**), ventrolateral (**d** and **f**) and dorsal (**e** and **g**) axons by Sema3A, Sema3F and Sema3E. Explants were obtained at E14.5 (**a–d**) or E16.5 (**e–h**), cultured for 2 days *in vitro* (DIV) and processed for βIII-tubulin (clone TUJ-1) immunostaining. **(a)** Dotted line defines the boundary between the proximal (P) and the distal (D) quadrant of the explants. Note the strong chemorepulsion of hippocampal axons by Sema3A at E14.5. In addition, Sema3E-mediated chemorepulsion can be seen on hippocampal, entorhinal and ventrolateral cortex at E14.5 (**b–d**). Examples of Sema3F-mediated chemorepulsion on ventrolateral axons and Sema3E-effects on hippocampal axons can be seen in **f** and **h** respectively. This contrasts with what is observed for dorsal neocortical axons (**g**). (**i**–**m**) Representative phalloidin-TRITC stained neuronal processes of cultured entorhinal explants from E16.5, illustrating semaphorin-mediated growth cone collapse. **(i)** Normal growth cones, with lamellipodia and filopodia (arrows) from entorhinal explants cultured with SEAP medium. (**j**–**l**) Examples of collapsed growth cones (asterisks) after incubation with Sema3A (**j**), Sema3E (**k**) and Sema3F (**l**). (**m**) Histogram illustrating percentages of collapsed growth cones per explant after the incubation of entorhinal explants with secreted semaphorins. Results represent the mean ± S.E.M. of three separate experiments. Asterisks indicate statistical differences between groups and controls. ***P ≤ 0.001; ANOVA Bonferroni *post hoc* test. Abbreviations: CA = CA1-3 hippocampal regions; EC = entorhinal cortex; DC = dorsal neocortex; VC = ventrolateral neocortex. Scale bars: **a** = 150 μm pertains to (**b**–**h**); **i** = 20 μm pertains to (**j**–**l**).

We aimed to explore whether these changes in mRNA expression patterns, especially of *Np1*, *Np2* and *PlxnD1*, correlated with particular chemorepulsive actions of secreted semaphorins at E14.5 and E16.5 embryonic stages. These stages correlate with the ingrowth of entorhinal axons in the hippocampus and with the main outgrowth of hippocampal axons (Fig. 1). A summary of the results obtained can be seen in Table 1 and representative examples of explants cultures can be seen in Fig. 2. As indicated, explants showing increased numbers of axons in the distal quadrant in comparison to the proximal (suggesting chemorepulsion) showed a proximal quadrant/distal quadrant (P/D) < 1, in contrast to radial outgrowth (P/D = 1) or chemoattractive (P/D > 1) responses (see^46^ and Supplementary Fig. 2a for details). Results indicated that Sema3E was able to induce chemorepulsion of CA1-3 (E14.5; ≈87.5%; P/D ratio < 1; n = 16), subiculum (E14.5; P/D ratio < 1; ≈75%; n = 16), entorhinal cortex (E14.5; P/D ratio < 1; ≈93.75%; n = 16), and ventrolateral (E14.5; P/D ratio < 1; ≈85.71%; n = 14) but not dorsal (parietal) neocortex axons at E14.5. (E14.5; ≈90.9%; P/D ratio = 1; n = 11). At later embryonic stages (E16.5) these chemorepulsive actions were largely maintained ((CA1-3; ≈75%; P/D ratio < 1; n = 16); (subiculum; ≈60%; P/D ratio < 1; n = 15) (ventrolateral neocortex; ≈56.25%; P/D ratio < 1; n = 16)) except for entorhinal cortex (E16.5; ≈41%; P/D ratio = 1; n = 22). These effects largely correlate with *PlxnD1* mRNA distribution (Fig. 1). Sema3E-induced chemorepulsive effect on entorhinal axons at E14.5 was analysed in explants derived from Nestin-cre; *PlxnD1*^flox/flox^ mice (Supplementary Fig. 2d). In this case, we observed that *Nestin-cre*; *PlxnD1*^*flox/flox*^ E14.5 entorhinal explants showed a drastic reduction in chemorepulsive response (≈54.5%; P/D ratio < 1; ≈45.5%; P/D ratio = 1; n = 22). In addition, Sema3A elicited clear chemorepulsive action on CA1-3 axons at E14.5 (≈71.4%; P/D ratio < 1; n = 7) with decreasing effects at E16.5 (≈42.8%; P/D ratio < 1; n = 14 and ≈42.8%; P/D ratio = 1; n = 14). In contrast, Sema3F-mediated chemorepulsion on CA1-3 axons increased from E14.5 (≈54.5%; P/D ratio < 1; n = 6) to E16.5 (≈100%; P/D ratio < 1; n = 11). For ventrolateral neocortex, Sema3F-mediated chemorepulsion was observed at E14.5 (≈63.6%; P/D ratio < 1; n = 11) but relevantly at E16.5 (≈100%; P/D ratio < 1; n = 14). As indicated above, we were unable to determine any chemorepulsive effect in dorsal neocortex at these stages (Fig. 2g; Table 1). With respect to entorhinal axons we observed that chemorepulsion mainly took place at E16.5 for Sema3F (≈77.7%; P/D ratio < 1; n = 18) and with a tendency for Sema3E (≈41%; P/D ratio < 1 and ≈50%; P/D ratio = 1; n = 22) and Sema3A (≈41.6%; P/D ratio < 1 and ≈50%; P/D ratio = 1; n = 12). These last results were corroborated in collapse experiments on identified growth cones of entorhinal cortex explants at E16.5 (Fig. 2i–m). All three semaphorins induced collapse of growth cones with the strongest effects induced by Sema3F (Fig. 2l,m) (e.g., Sema3F; 66.94 ± 3.68; *vs* SEAP; 28.95 ± 6.210; Mean ± S.E.M.; *t* = 4.954; *P* = 0.0005; confidence interval 95% = −60.57 to −15.42; ANOVA, Bonferroni *post hoc* test).

**Table 1:** Effects of Sema3A, Sema3F and Sema3E on the growth of CA1–3, entorhinal, subicular, ventrolateral and dorsal cortical axons in confrontation experiments (see Material and methods for details).

E14.5	CA			SUB			EC			VC			DC		
P/D	<1	1	>1	<1	1	>1	<1	1	>1	<1	1	>1	<1	1	>1
S3A	5 (71.4%)	1 (14.3%)	1 (14.3%)	2 (18.18%)	2 (18.18%)	7 (63.63%)	3 (23.08%)	4 (30.77%)	6 (46.15%)	4 (30.77%)	8 (61.54%)	1 (7.69%)	4 (25%)	9 (56.25%)	3 (18.75%)
S3F	6 (54.54%)	2 (18.18%)	3 (27.27%)	2 (14.28%)	8 (57.14%)	4 (28.57%)	3 (21.43%)	7 (50%)	4 (28.57%)	7 (63.63%)	3 (27.27%)	1 (9.1%)	6 (40%)	6 (40%)	3 (20%)
S3E	14 (87.5%)	2 (12.5%)	0 (0%)	12 (75%)	4 (25%)	0 (0%)	15 (93.75%)	1 (6.25%)	0 (0%)	12 (85.71%)	2 (14.29%)	0 (0%)	0 (0%)	10 (90.9%)	1 (9.1%)
SEAP	4 (66.66%)	2 (33.33%)	0 (0%)	5 (71.43%)	2 (28.57%)	0 (0%)	2 (66.66%)	1 (33.33%)	0 (0%)	2 (66.66%)	1 (33.33%)	0 (0%)	3 (37.5%)	5 (62.5%)	0 (0%)
**E16.5**	**CA**			**SUB**			**EC**			**VC**			**DC**		
P/D	<1	1	>1	<1	1	>1	<1	1	>1	<1	1	>1	<1	1	>1
S3A	6 (42.85%)	6 (42.85%)	2 (14.3%)	10 (66.66%)	5 (33.33%)	0 (0%)	5 (41.66%)	6 (50%)	1 (8.33%)	4 (33.33%)	6 (50%)	2 (16.67%)	3 (23.08%)	7 (53.84%)	3 (23.08%)
S3F	11 (100%)	0 (0%)	0 (0%)	9 (60%)	5 (33.33%)	1 (6.67%)	14 (77.7%)	3 (16.67%)	1 (5.56%)	14 (100%)	0 (0%)	0 (0%)	2 (18.18%)	6 (54.54%)	3 (27.27%)
S3E	12 (75%)	4 (25%)	0 (0%)	9 (60%)	6 (40%)	0 (0%)	9 (40.9%)	11 (50%)	2 (9.1%)	9 (56.25%)	7 (43.75%)	0 (0%)	4 (33.33%)	3 (25%)	5 (41.67%)
SEAP	3 (30%)	7 (70%)	0 (0%)	5 (50%)	4 (40%)	1 (10%)	1 (10%)	7 (70%)	2 (20%)	3 (33.33%)	4 (44.44%)	2 (22.23%)	3 (42.86%)	4 (57.14%)	0 (0%)

The effects of these class III secreted semaphorins on the experiments are summarized as P/D < 1, P/D = 1 or P/D > 1, indicating chemorepulsion, radial outgrowth or chemoattraction, respectively. For each region the number of cultures displaying these effects is shown. Abbreviations: CA = CA1-3 hippocampal regions; D = distal quadrant; DC = dorsal cortex; EC = entorhinal cortex; P = proximal quadrant; SUB = subiculum; VC = ventrolateral cortex.

### Altered pattern of the EH connection in absence of PlexinD1 and Sema3E

Since *PlxnD1*^*0/0*^ mice die prenatally, we crossed *PlxnD1*^*flox/flox*^ with *Nestin-cre* mice. In the resulting conditional mutant *Nestin-cre*; *PlxnD1*^*flox/flox*^, the decrease of *PlxnD1* mRNA was corroborated histologically by *in situ* hybridization at P0 (Supplementary Fig. 3b). As Nestin promoter is active from E12-E13, early-generated hippocampal Cajal-Retzius cells were labelled by *PlxnD1* probes at P0. In addition, blood vessels were also labelled with *PlxnD1* probes in some brain regions (Supplementary Fig. 3b). Absence of *PlxnD1* did not alter *Sema3E*, *Np1* or *Np2* (Supplementary Fig. 3c–f) or *PlxnA1* expression (not shown).

**Figure 3:**
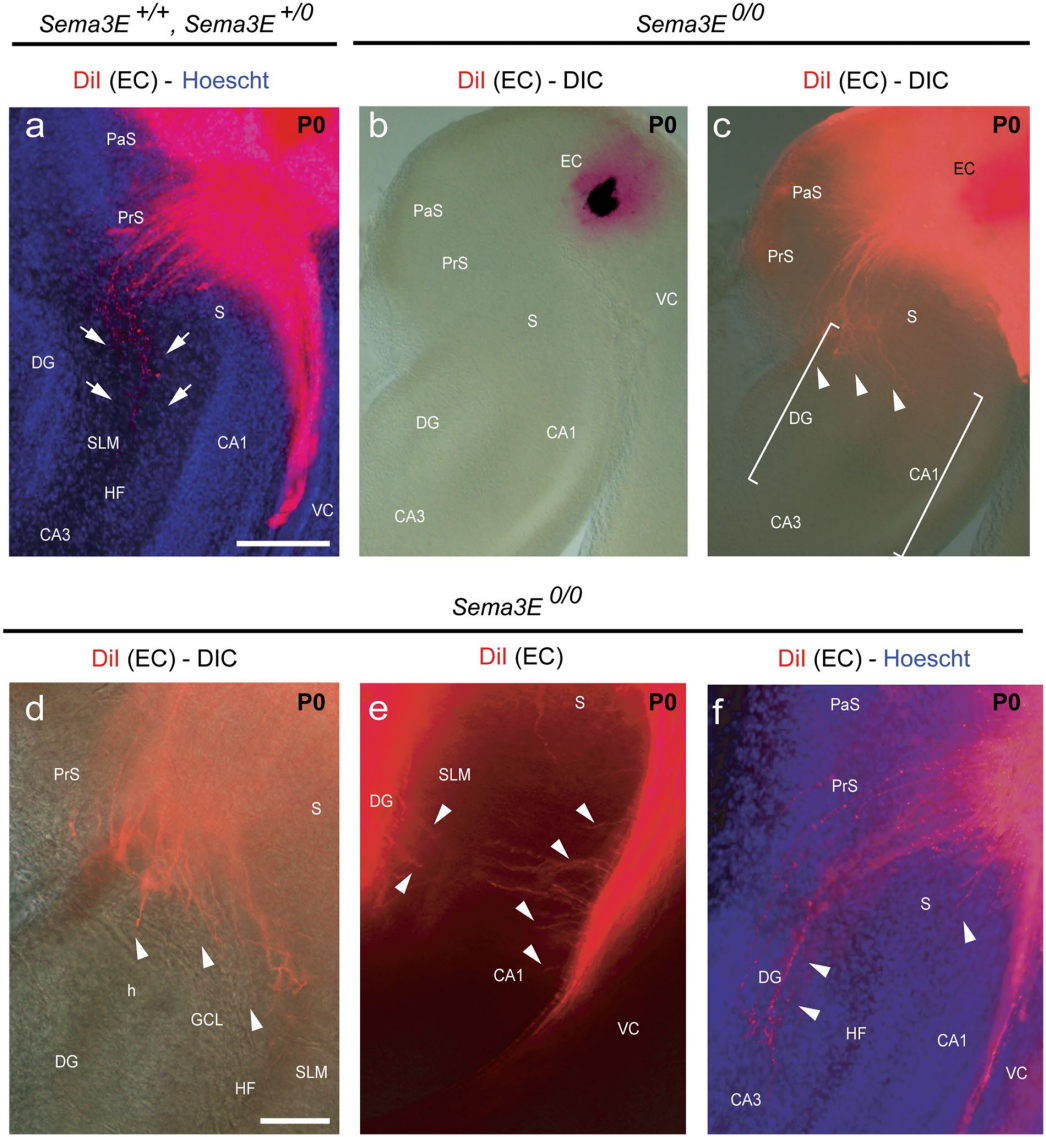
Pattern of entorhino-hippocampal innervations in Sema3E-deficient mice after DiI injections in the entorhinal cortex at P0. (**a**) In wild-type and *Sema3E*^+*/0*^ mice, entorhinal fibers are restricted to the stratum lacunosum-moleculare (arrows) and the white matter. (**b**–**f**) In *Sema3E*^*0/0*^ mice the EH connection is formed, but ectopic axons cross the hippocampal fissure, entering the dentate gyrus or the CA1 (arrowheads in **b**–**f**). Abbreviations as in Fig. 1 and GCL = granule cell layer; h = hilus; HF = hippocampal fissure; PrS = presubiculum; SLM = stratum lacunosum- moleculare. Scale bar: **a** = 250 μm pertains to (**b**–**c**); **d** pertains to (**e**–**f**) = 100 μm.

Organotypic slices of the hippocampal formation have been used largely to determine the development and layer specific targeting of entorhinal^13,47^ septal^29^ and commissural connections^19^ in several experimental conditions. Thus, taking advantage of this culture preparation, we established EH organotypic slice cultures from newborn mutant *Nestin-cre*; *PlxnD1*^*flox/flox*^ mice (Supplementary Fig. 3g,h). After byocitin tracing, the entorhino-hippocampal connection was formed and entorhinal axons were able to cross the subiculum, reaching the hippocampal slm/ml in *Nestin-cre*; *PlxnD1*^*flox/*+^ *and Nestin-cre*; *PlxnD1*^*flox/flox*^ entorhino-hippocampal cocultures (Supplementary Fig. 3g,h). However, numerous byocitin-positive misrouted fibers were seen in the CA1 and in the DG (red asterisks in Supplementary Fig. 3h) of *Nestin-cre*; *PlxnD1*^*flox/flox*^ cultures compared to their control littermate *Nestin-cre*; *PlxnD1*^*flox/*+^ (Supplementary Fig. 3g). Next we traced the EH connection with the anterograde lipophilic tracer DiI at P0 in *Sema3E*^*0/0*^ and control mice (Fig. 3). In control (wild-type and *Sema3E*^+*/0*^) mice, DiI axons followed the perforant pathway, being restricted to the slm (arrows in Fig. 3a) and the white matter. In contrast, in *Sema3E*^*0/0*^ mice (Fig. 3b–f) the EH connection was formed, but numerous axons crossed the hippocampal fissure, perforating the granule cell layer and reaching the dentate hilus (arrowheads in Fig. 3c–f). In addition, large numbers of DiI-labeled ectopic axons were also observed crossing the subiculum-CA1 from the hippocampal alveolar path towards the slm (arrowheads in Fig. 3c–f). These effects were seen in all mutant mice (n = 10) from three different litters of *Sema3E*^*0/0*^ mice. In other words, the absence of Sema3E or PlexinD1 led to misrouted entorhinal axons during the establishment of the entorhino-hippocampal connection.

### Increased proliferation and ectopic granule cells in absence of *Sema3E* or *PlxnD1*

**Figure 4:**
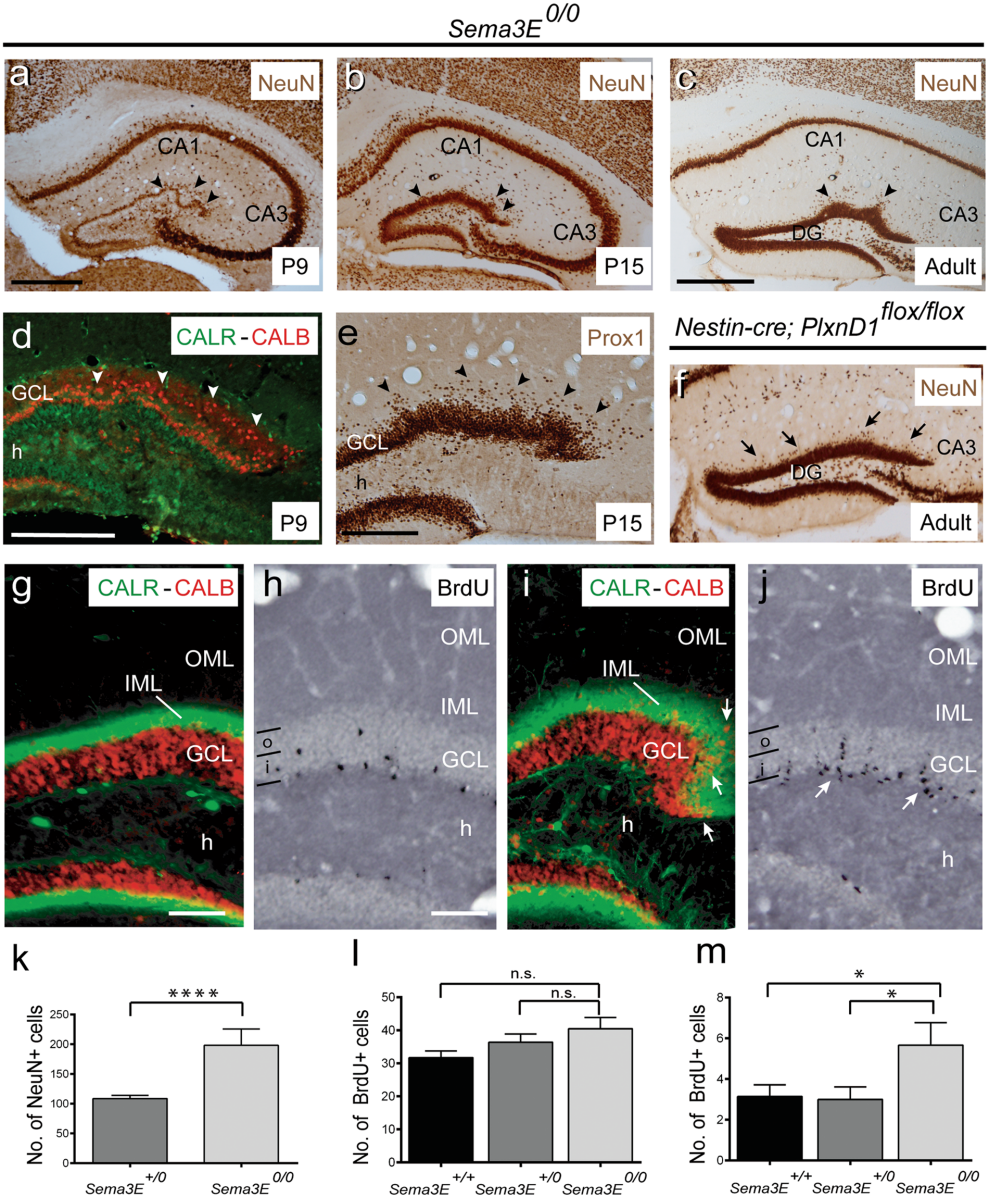
Examples of α-NeuN immunostaining in the hippocampus proper and dentate gyrus of *Sema3E*^*0/0*^ (**a**–**c**) and *Nestin-cre*; *PlxnD1*^*flox/flox*^ (**f**) mice at different postnatal stages. Note the presence of numerous NeuN-positive cells in the molecular layer in absence of Sema3E/PlexinD1 signalling (arrowheads), especially in Sema3E-deficient mice. Also note the presence of several waves of the suprapyramidal blade of dentate gyrus. (**d**) Double immunolabeling of Calretinin (green) and Calbindin (red) in the dentate gyrus of *Sema3E*^*0/0*^ mice at P9. Note the presence of numerous ectopic Calbindin-positive neurons in the IML of *Sema3E*^*0/0*^ mice (arrowheads). (**e**) Prox-1 immunolabeling in the dentate gyrus of *Sema3E*^*0/0*^ mice at P15 shows the presence of numerous Prox-1-positive granule cells in the molecular layer (arrowheads). (**g**,**i**) Double immunolabeling of Calretinin (green) and Calbindin (red) in the dentate gyrus of control (**g**) and *Sema3E*^*0/0*^ adult mice (**i**). Note the presence of numerous ectopic Calbindin-positive neurons in the IML of *Sema3E*^*0/0*^ mice (arrows in **i**). (**h**–**j**) Examples of BrdU-labeled neurons in the granule cell layer of control (**h**) and *Sema3E*^*0/0*^ (**j**) mice. Numerous BrdU-positive cells forming columns in the granule cell layer can be seen in mutant mice (arrows in **j**). (**k**) Histogram illustrating the number of NeuN-positive cells in the IML of the suprapyramidal blade of the dentate gyrus in *Sema3E*^+*/0*^ *and Sema3E*^*0/0*^ mice. (**l**–**m**) Histograms illustrating the number of BrdU-positive cells counted in the whole granule cell layer (**l**) and in the outer portion of the granule cell layer of the dentate gyrus (labeled as ‘o’) (**m**) in *Sema3E*^+*/*+^, *Sema3E*^+*/0*^ *and Sema3E*^*0/0*^ mice. Asterisks indicate statistical differences between groups. *P ≤ 0.05; **P ≤ 0.01; ***P ≤ 0.001; ****P ≤ 0.0001. ANOVA Bonferroni *post hoc* test. Abbreviations as in Figs 1–3 and i = inner portion of the granule cell layer; IML = inner molecular layer; o = outer portion of the granule cell layer and OML = outer molecular layer. Scale bars: **a** = **b** = 500μm; **c** = **f** = 500 μm; **d** = 200 μm; **e** = 200 μm; **g** and **h** = 150 μm pertains to **i** and **j** respectively.

**Figure 5:**
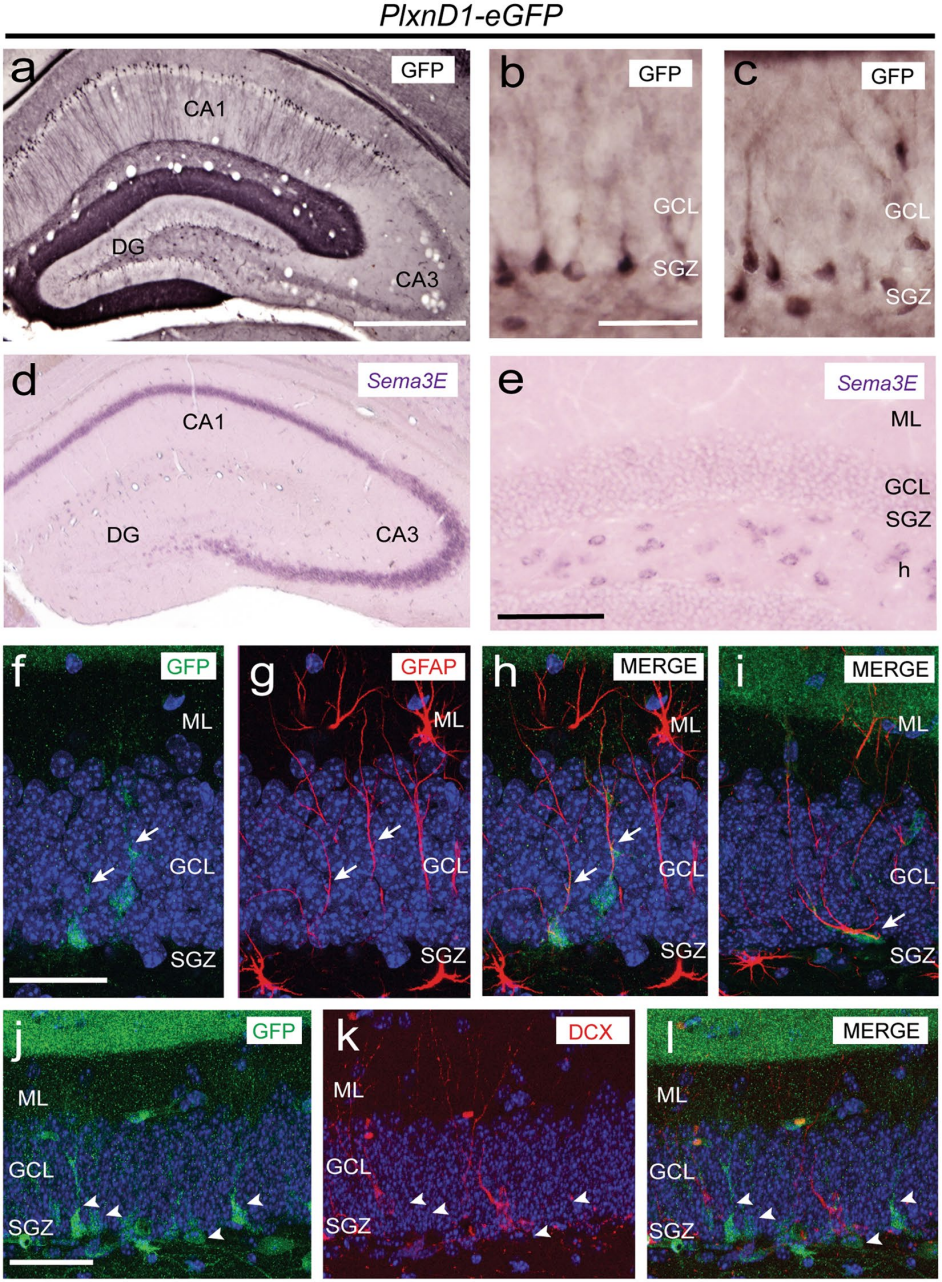
Low- **(a**) and high- (**b,c**) power photomicrographs illustrating GFP-positive neurons in the hippocampus (**a**) and dentate gyrus (**b,c**) of adult PlxnD1-eGFP mice. Note GFP labelling in subsets of pyramidal neurons CA1 and cells of hilus and subgranular zone. Low- (**d**) and high- (**e**) power photomicrographs illustrating the distribution of *Sema3E* mRNA in the hippocampus (**d**) and dentate gyrus (**e**) of adult wild-type mice. (**f**–**i**) Confocal immunofluorescence images for GFP (green) and GFAP (red) in the dentate gyrus of adult *PlxnD1-eGFP* mice. Note that all GFP-positive cells express GFAP marker (arrows). (**j**–**l**) Confocal immunofluorescence images for GFP (green) and DCX (red) in the dentate gyrus of adult *PlxnD1-eGFP* mice. Note that not all GFP-positive cells express DCX marker (arrowheads). Abbreviations as in Figs 1–4 and ML = molecular layer; SGZ = subgranular zone. Scale bars: **a** = 500 µm pertains to **d**; **b** = 50 µm pertains to **c**; **e** = 150 µm; **f** = 50 µm pertains to (**g**–**i**); **j** = 50 µm pertains to (**k**,**l**).

**Figure 6:**
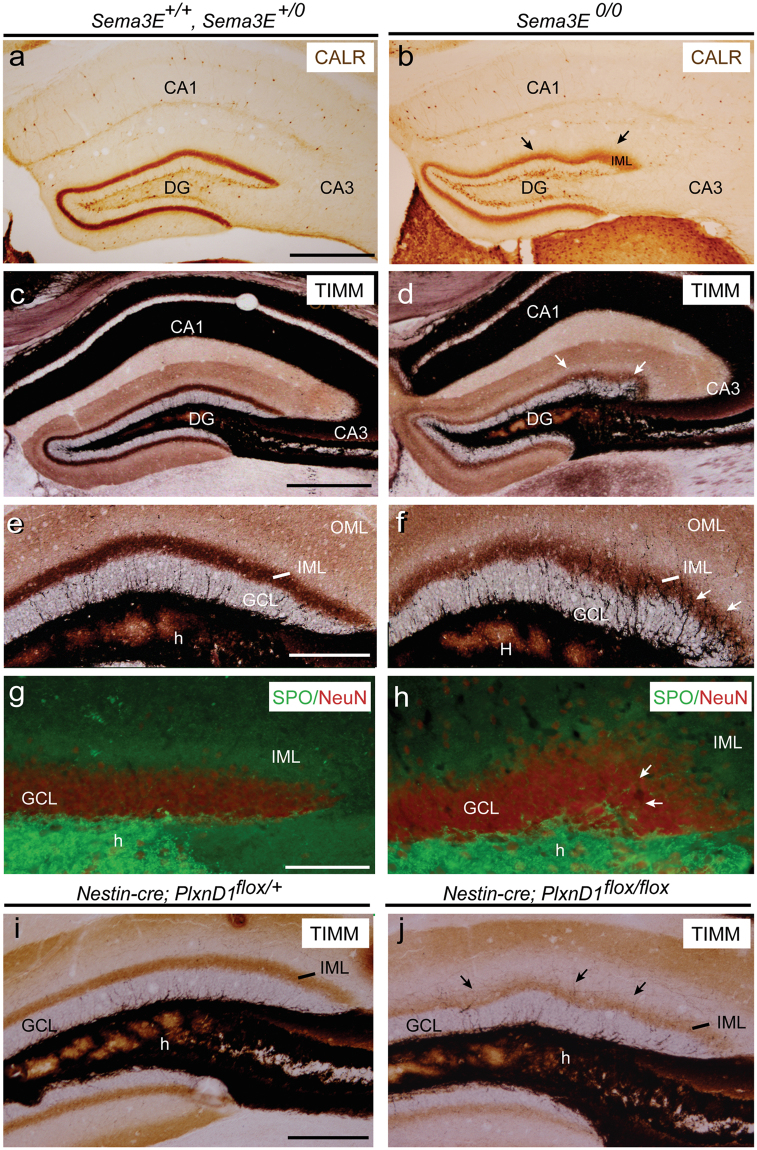
(**a,b**) Examples of α-Calretinin immunostaining in the hippocampus proper and dentate gyrus of adult *Sema3E*^+*/*+^ *and Sema3E*^+*/0*^ (**a**) and *Sema3E*^*0/0*^ (**b**) mice. Note the presence of several waves in the IML of the suprapyramidal blade (arrows in **b**). (**c–f**) Photomicrographs illustrating the pattern of selenite-silver staining (TIMM) in *Sema3E*^+*/0*^ *and Sema3E*^+*/*+^ (**c**,**e**) and *Sema3E*^*0/0*^ (**d**,**f**) mice. Note the numerous ectopic mossy fibers crossing the granule cell layer entering the molecular layer of mutant mice (arrows in **d**,**f**). (**g**–**h**) Double immunolabeling of Synaptoporin (SPO, green) and NeuN (red) in the dentate gyrus of *Sema3E*^+*/*+^ *and Sema3E*^+*/0*^ (**g**) and *Sema3E*^*0/0*^ (**h**) mice showing ectopic mossy fibers crossing the granule cell layer in mutant mice (arrows in **h**). (**i**–**j**) High-power photomicrographs illustrating the pattern of selenite-silver staining (TIMM) in control (*Nestin-cre*; *PlxnD1*^*flox/*+^) and PlexinD1-deficient (*Nestin-cre*; *PlxnD1*^*flox/flox*^) mice. Abbreviations as in Figs 1–5 and CALR = calretinin; SPO = synaptoporin. Scale bar: **a** = 500 μm pertains to **b**; **c** = **d** = 500 μm. **e** = 150 μm pertains to (**f**,**i**–**j**); **g** = **h** = 100 μm.

To further investigate whether absence of Sema3E/PlexinD1 signalling could lead to other abnormalities, we processed brain sections from different postnatal (P9 and P15) and adult stages to check the cytoarchitecture of the hippocampal formation (Figs 4 and 5) and putative changes in axonal connections (Fig. 6). In histological analysis from P9 onwards, hippocampus from *Sema3E*^*0/0*^ showed an unshaped dentate gyrus with the presence of several waves of the suprapyramidal blade of dentate gyrus (arrowheads in Fig. 4a–c) and with a great number of ectopic NeuN-positive cells in the molecular layer (Fig. 4k) *(Sema3E*^*0/0*^; 197.9 ± 7.38; *vs Sema3E*^+*/0*^; 108.7 ± 5.49; Mean ± S.E.M.; *t* = 9.699; *P* < 0.0001; confidence interval 99% = 63.43 to 115.1; unpaired t test with Welch’s correction, ****P ≤ 0.0001). These changes were also seen, although less pronounced, in adult *Nestin-cre*; *PlxnD1*^*flox/flox*^ mice (Fig. 4f). Presence of misallocated granule cells was validated with different staining such as double immunofluorescence for Calretinin-Calbindin (Fig. 4d,g,i) or single immunostaining of Prox-1(Fig. 4e). Double-labelled (Calretinin-Calbindin) sections illustrated the presence of ectopic Calbindin-positive cells outside the granule cell layer crossing the inner molecular layer (iml) of the suprapyramidal blade in *Sema3E*^*0/0*^ mice at all stages analysed (Fig. 4d,i). Next, we aimed to determine the cause of the abnormal distribution of granule cells in the hippocampus. To this end, we intraperitoneally injected adult mice with BrdU for 4 days and, one week later, mice were processed for immunohistochemistry. Cell counts increased, although this was not statistically significant, for the numbers of BrdU-positive cells in the granule cell layer of *Sema3E*^*0/0*^ mice (Fig. 4l). BrdU-immunoreacted sections also showed numerous BrdU-positive cells at different levels of the granule cell layer, often forming columns spanning the layer in *Sema3E*^*0/0*^ mice (arrows in Fig. 4j). Indeed, we detected an increase in BrdU-positive cells in the outer portion of the granule cell layer of *Sema3E*^*0/0*^ compared to *Sema3E*^+*/0*^ and *Sema3E*^+*/*+^ mice (Fig. 4h,j,m) (*Sema3E*^*0/0*^; 5.67 ± 1.11; *vs Sema3E*^+*/0*^; 3.0 ± 0.61; Mean ± S.E.M.; *t* = 2.291; *P* = 0.0849; confidence interval 90% = −5.248 to −0.084; *Sema3E*^*0/0*^; 5.67 ± 1.11; *vs Sema3E*^+*/*+^ mice; 3.14 ± 0.57; *t* = 2.301; *P* = 0.083; confidence interval 90% = −4.957 to −0.09061; ANOVA, Bonferroni *post hoc* test, *P < 0.1). The presence of different immature cells in different locations of the granule cell layer was also observed with electron microscopy techniques (Supplementary Fig. 4). In these sections, both ectopic granule cells in the molecular layer and immature cells located in cell niches of the subgranular layer were clearly observed (Supplementary Fig. 4a,b). *Sema3E*^*0/0*^ mutant mice exhibited a larger number of cell niches with more immature cells within them. As a consequence, the subgranular contour of these animals is more irregular when compared to the same region in *Sema3E*^+*/0*^ animals (Supplementary Fig. 4a–d). In parallel, due to the relevance of the vascular system in stem cell niche biology we aimed to determine changes in vasculature in the hippocampus of *Sema3E*^*0/0*^ mice. However, the analysis of blood vessel distribution in the hippocampus revealed no relevant alterations between control and mutant mice (Supplementary Fig. 5). In conclusion, absence of Sema3E led to increased proliferation of the subgranular zone precursors of the adult dentate gyrus.

### PlexinD1 is expressed in type-1 radial glia-like stem cells

After the description of the dysregulation of cell proliferation in subgranular cells, we next explored whether Sema3E/PlexinD1 signalling could influence adult neurogenesis. First, we determined PlexinD1 expression in the adult hippocampus of *PlxnD1*-*eGFP* mice using GFP antibody and we observed cellular PlexinD1 labelling in subsets of pyramidal CA1neurons and in cells from the hilus and subgranular zone (Fig. 5a–c) suggesting that it could be expressed by proliferating cells. The presence of PlexinD1-expressing cells in the dentate gyrus has also been reported in^48^ and published on the Allen Brain Atlas web site (http://mouse.brain-map.org, experiment 73521005). Additionally, *Sema3E* mRNA expression was detected in the pyramidal layer and the *stratum lacunosum-moleculare* of CA1-3 and in the hilus with lesser presence in the granule cell layer (Fig. 5d,e). To identify cell types expressing GFP in the subgranule zone of *PlxnD1*-*eGFP* mice we performed double immunofluorescence with different neurogenic markers and GFP. Double-labelled (GFP/GFAP) sections illustrated that GFP was present in numerous type-1 radial GFAP-positive cells in the subgranular zone (Fig. 5f–i) but absent in immature neurons (DCX-positive neurons) (Fig. 5j–l), leading us to conclude that PlexinD1 is present in radial type-1 glia-like stem cells in the subgranular zone.

### Aberrant mossy fiber distribution in absence of Sema3E/PlexinD1 signalling

Next we analysed the distribution of the hippocampal connections in adult mice lacking Sema3E/PlexinD1 signalling (Fig. 6). BDA labelling of EH axons in adult *Sema3E* ^*0/0*^ mice revealed no relevant changes in the distribution of labelled entorhinal axons in the CA1-3 regions and layer (slm/ml) or in the wavy distribution of the EH axons in the dentate molecular layer (data not shown). In parallel, sections of Sema3E-deficient mice immunostained with α-Calretinin antibody (to label commissural-associational connections in the dentate molecular layer) showed diffuse puncta-like labelling in the inner portion of the dentate molecular layer compared to *Sema3E*^+*/*+^ and *Sema3E*^+/0^ mice (Fig. 6a,b). Calretinin-positive labelling in the iml was apposed to the wavy granule cell layer (Fig. 6b). Parallel sections processed for Calbindin immunostaining showed that the coarse distribution of the mossy fiber projections (supra and infrapyramidal) was similar in mutant and control mice (not shown).

However, after selenite-silver staining for zinc-rich projections we observed profound changes in the mossy fiber distribution in the dentate gyrus of the *Sema3E*^*0/0*^ mice with numerous ectopic fibers in the iml, especially in the gyri of the waves reaching the oml (Fig. 6c–f). Mossy fiber sprouting was further corroborated in parallel sections immunoprocessed for the detection of the presynaptic marker Synaptoporin (SPO) (Fig. 6g,h). SPO-immunoreacted sections revealed that some mossy fibers sprouted into the granule cell layer of Sema3E-deficient mice (Fig. 6h). This aberrant mossy fiber distribution was observed in all the mutant animals analysed from four different *Sema3E*^*0/0*^ litters.

Finally, we analysed sections from *Nestin-cre*; *PlxnD1*^*flox/flox*^ mice in order to detect changes in the mossy fiber distribution. As observed in the absence of Sema3E, mice lacking PlexinD1 (*Nestin-cre*; *PlxnD1*^*flox/flox*^) also showed aberrant sprouting into the granule cell layer compared to their control littermates *(Nestin-cre*; *PlxnD1*^*flox/*+^) (Fig. 6i,j). In conclusion, mice lacking either Sema3E or PlexinD1 showed relevant changes in the layer patterning of hippocampal mossy fibers.

### Dysregulation of excitability in *Sema3E*^*0/0*^ hippocampus

**Figure 7:**
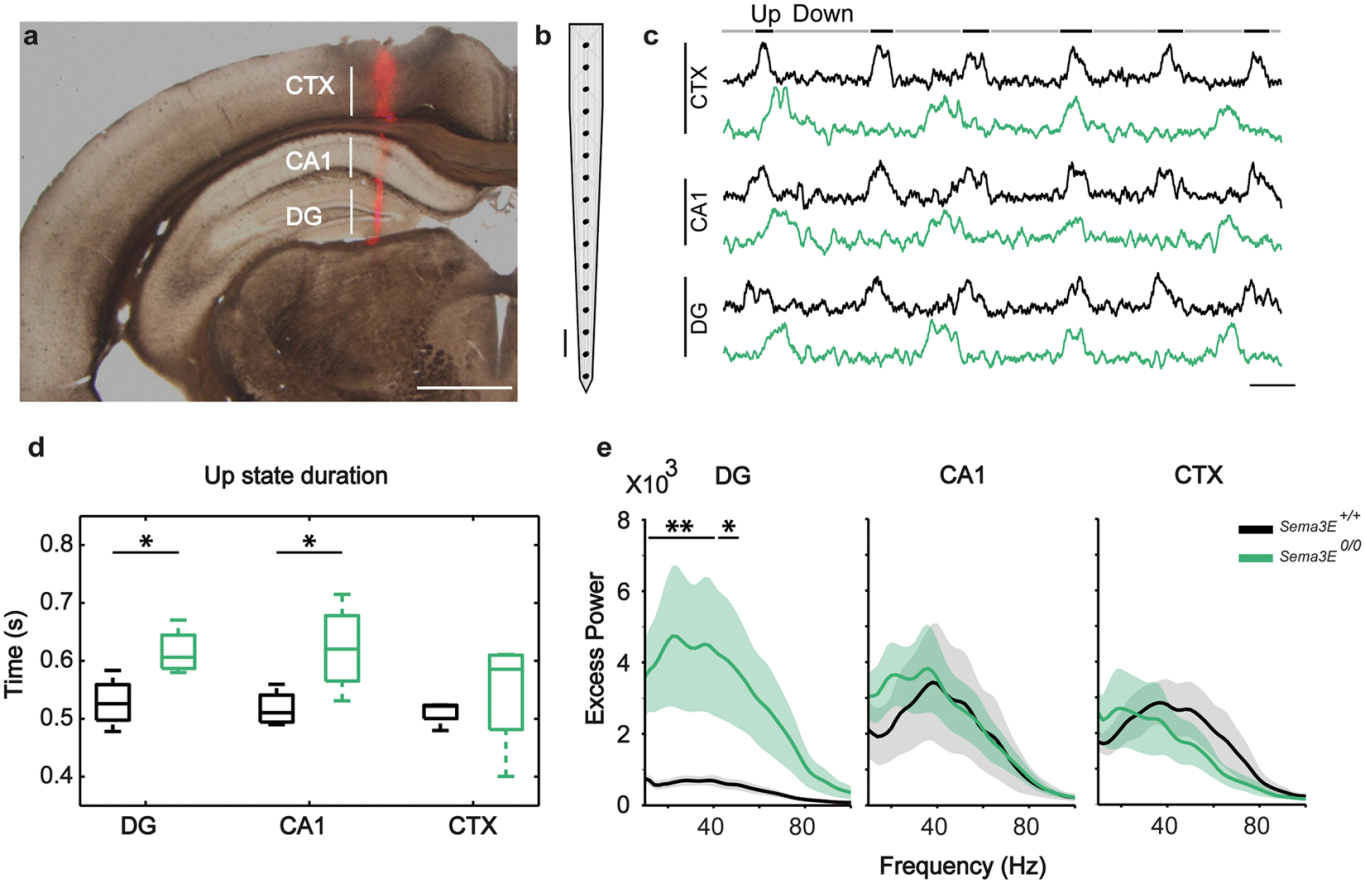
Alterations in the spontaneous oscillatory activity in *Sema3E*^*0/0*^ mice. (**a)** Representative half coronal section (2.0 mm posterior to Bregma, 1.0 mm lateral from midline) showing the track of a 1 × 16 multichannel recording probe covering the cerebral cortex (CTX) and hippocampus (CA1 and DG) of a mouse. Track reconstruction was accomplished following the tissue deposition of the DiI that was applied to probe prior to insertion. (**b**) Schematic representation of the 16-multichannel recording probe used to record neuronal activity. (**c**) Representative examples showing multi-unit activity traces (200–1500 Hz) simultaneously recorded in the cerebral cortex (CTX) and hippocampus (CA1 and DG) of a *Sema3E*^+*/*+^ (black) and a *Sema3E*^*0/0*^ (green) mouse during slow oscillatory activity. No vertical scale because they are arbitrary units (see materials and Methods). (**d**) Up state durations recorded in the hippocampus (DG and CA1) and cerebral cortex (CTX) of *Sema3E*^+*/*+^ (black, n = 4) and *Sema3E*^*0/0*^ (green, n = 4) mice. Box plots represent the first and third quartiles with the median depicted by the horizontal line within the box and extreme values shown by whiskers. (**e**) Average excess power (ratio between the mean power spectral density and the fit of the 1/f decay) during local field potential Up states recorded in the hippocampus (DG and CA1) and cerebral cortex (CTX) of *Sema3E*^+*/*+^ (black, n = 4) and *Sema3E*^*0/0*^ (green, n = 4) mice. Data expressed as mean ± S.E.M (shadow). *P < 0.1, **P < 0.05, Wilcoxon rank-sum test. Scale bar: **a** = 1 mm; **b** = 100 μm; **c** = 0.5 s.

The presence of aberrant synaptic connections in the dentate gyrus has been described in several models of epilepsy^49,50^. The unveiling of ectopic mossy fiber terminals in the dentate gyrus of *Sema3E*^*0/0*^ mice opened the question of whether the animals would functionally show epileptic-like activity patterns or enhanced neural excitability. To answer this, we recorded spontaneous local field potential (LFP) activity by means of a 16-channel multi-electrode array (100 µm spacing) covering the DG and CA1 areas of the hippocampus and the overlying neocortex (Fig. 7a,b) in a cohort of 8 mice (4 *Sema3E*^*0/0*^ and 4 *Sema3E*^+*/*+^) under deep anesthesia (see Materials and methods for details). Under these conditions, the brain activity is dominated by the characteristic dynamics of the slow-wave oscillatory activity (Fig. 7c), consisting of periods of high-firing activity called Up states, interspersed with almost silent epochs known as Down states^51–53^. Careful observation of the LFP profile revealed no evidence of epileptiform activity patterns in either group of animals (data not shown). Furthermore, in a subset of 3 *Sema3E*^+*/*+^ and 3 *Sema3E*^*0/0*^ animals, administration of the epileptogenic kainic acid (10 mg/kg, ip) revealed no differences in the dynamics of epileptic epochs between genotypes. Neither the latency to first discharge (*Sema3E*^*0/0*^: 2400 ± 400 s, *Sema3E*^+*/*+^: 1800 ± 600 s) nor the frequency of epileptic epochs (*Sema3E*^*0/0*^: 0.03 ± 0.02 Hz, *Sema3E*^+*/*+^: 0.02 ± 0.01 Hz) or their duration (*Sema3E*^*0/0*^: 27 ± 3 s, *Sema3E*^+*/*^: 31 ± 11 s) were significantly different between *Sema3E*^*0/0*^ and *Sema3E*^+*/*+^ animals (P > 0.05, Wilcoxon rank-sum test for all comparisons). Therefore, animals lacking Sema3E not only lacked spontaneous epileptic discharges, but they also lacked an enhanced tendency to express epileptiform activity patterns. Next, the spontaneous physiological activity, characterized by a slow oscillatory signal, was quantified by measuring parameters of the Up and Down states as reported in other mouse models^52^. Analysis of the multiunit activity revealed a trend towards the elongation of Up states in the CA1 and dentate gyrus of *Sema3E*^*0/0*^ (Fig. 7c,d, green) with respect to *Sema3E*^+*/*+^ (Fig. 7c,d, black) animals (*P* < 0.1, Wilcoxon rank-sum test), but not at the level of the overlying neocortex (Fig. 7c,d). Up states are generated by recurrent connectivity of local circuits and their duration is influenced by the balance between excitation and inhibition^54–56^ so elongated Up states have been considered a measure of increased excitability^57,58^. The longer duration of Up states at the level of dentate gyrus and CA1 areas of the hippocampus on *Sema3E*^*0/0*^ animals suggests an increased network excitability.

A fine balance between excitation and inhibition is also required for the generation of higher-frequency oscillatory components in both cortex and hippocampus^54,59^. In particular this gamma oscillatory activity, prominent in the aroused brain, has been implicated in higher-level cortical processes, such as sensory binding, storage of memories, and consciousness^60^ (see also^61^ for review on hippocampal-associated gamma oscillations). Thus, to gain further insight into the possible dysregulation of the excitatory-inhibitory balance in *Sema3E*^*0/0*^ animals, we computed the power of the recorded oscillatory activity at higher frequencies (10–100 Hz) in our three target recording areas: neocortex, CA1 and dentate gyrus. As shown in Fig. 7e, the power of high-frequency oscillatory activity around the low-gamma range (20–55 Hz) was significantly greater in the dentate gyrus of *Sema3E*^*0/0*^ animals compared to *Sema3E*^+*/*+^ (p < 0.05, Wilcoxon rank-sum test), while there were no significant differences at the level of either CA1 or cerebral cortex (*P* > 0.05, Wilcoxon rank-sum test).

## Discussion

### Role of Sema3E/PlexinD1 signalling during the development of hippocampal connections

The participation of secreted semaphorins during cortical wiring has been reported in numerous studies (see Introduction for references). The present study expands current knowledge with the description of the particular actions of Sema3E and its receptor PlexinD1 during the formation of the EH connection. The entorhinal cortex plays a crucial role as a gateway, connecting the neocortex and the hippocampal formation. Layers II and III of the entorhinal cortex give rise to the perforant pathway, the main source of excitatory input to the hippocampus^5–8^.

In the developing telencephalon, Sema3E/PlexinD1 functions have been described for Cajal-Retzius cell migration in neocortex^41^, as well as the development of subicular^31,32^ and striatal^14^ connections. However, their participation in the development of the EH connection had not been explored. Present results indicate that Sema3E is able to induce chemorepulsive actions on entorhinal, hippocampal and ventrolateral neocortical axons at E14.5 and E16.5. In a previous study^32^, Chauvet *et al*. reported that the addition of Sema3E to control cortical neurons from lateral (entorhinal-ventrolateral region in the present study) cortex (at E17.5) led to a 40% decrease in mean axonal length. Similar effects on cortical axons were also described by Deck *et al*. at E14.5–E16.5^36^. The present study, which indicates a similar chemorepulsive action on entorhinal axons growing in explant cultures and in confrontation assays (see Table 1) is in line with the above-mentioned study. In addition, our results demonstrate that this inhibition is PlexinD1 expression- dependent.

Our experiments also show chemorepulsive effects of Sema3E from E14.5 to E16.5 in hippocampal, entorhinal and ventrolateral neocortical axons, without chemoattractive responses. However, Bellon *et al*. reported Sema3E-induced chemoattraction on subicular neurons at E17.5^31^. This chemoattractive effect is VEGFR2-dependent^31^. In our study, we were unable to observe VEGFR2 expression in subicular neurons between E14.5–E16.5 by histological methods (not shown). However, the inhibitory effects of Sema3E in subiculum decreased from E14.5 to E16.5 (P/D >1 from 75 to 60%; with P/D = 1 from 25 to 40% respectively). Thus, it is reasonable to consider a gradual shift in Sema3E-elicited effects in subicular neurons between E14.5 and E17.5 that might correlate with increased VEGFR2 expression in projecting subicular neurons. In addition, changes in semaphorin sensitivity in entorhinal cortex explants were also observed in Sema3F from E16.5 onwards, supported by the increasing Np2 labeling in entorhinal neurons between E14.5 and E16.5 in the region.

Previously reported data of Sema3E included a unique chemorepulsive effect on CA1–3 axons only at E14.5 without any effect in entorhinal axons prenatally or in CA1-3 axons between E16.5 and P0^30^. In our study, Sema3E induced chemorepulsion in CA1-3 axons at E14.5 and E16.5, and in entorhinal axons at E14.5 with decreased effects at E16.5. In addition, ventrolateral cortical axons were also repelled by Sema3E at E14.5 and E16.5. In contrast, Sema3E is unable to trigger any apparent response in dorsal neocortex. Our data are strongly supported by previously published data^31^ and also correlate with the temporal evolution of PlexinD1 expression in different subsets of projecting neurons, although a complete mechanistic understanding of these effects is not yet available. Lastly, in our study we described the almost complete absence of all three semaphorins (A, F and E) in the subiculum compared to other hippocampal subfields. As the EH connection crossed the subicular region (perforant pathway) during its navigation towards the hippocampus, it is reasonable to believe that this low semaphorin expression constitutes an ‘axonal corridor’ for entorhino-hippocampal axons to ensure appropriate establishment of the EH connections during embryonic development, as is also indicated for thalamo-cortical connections^62^. Our tracing experiments determined that the absence of Sema3E signaling did not essentially alter the basic pattern of termination of EH axons *in vivo* at early postnatal stages, although it caused minor targeting errors as also reported in *Sema3A*^*0/0*^ mice^30^. These defects are not observed in adult hippocampus; they are probably resolved during postnatal synapse refinement as also reported in other axonal tracks of Sema3E*-*deficient mice^31,32^.

### Adult functions of Sema3E/PlexinD1 signalling in hippocampus

The first description of *Sema3E*^*0/0*^ mice included reduced anxiety levels and moderately impaired spatial working memory^32^. It is well known that changes in mossy fiber distribution in different mouse strains correlated with deficits in spatial and non-spatial memory tasks^63,64^. Since *Sema3E* ^*0/0*^ mutant mice showed changes in mossy fiber layer distribution, we cannot rule out the possibility that the described alterations also play a role in the impairment in the modest spatial tasks described in mutant mice^32^. The participation of several semaphorins (e.g., Sema3F^33^, Sema6A and Sema6B^65^ and their receptors (e.g., Neuropilin2^35^, PlexinA3^34^ or PlexinA4^65^) in mossy fiber development has also been described. Our data led us to suggest the participation of Sema3E in mossy fiber development in combination with both other semaphorins and other families of guidance molecules (e.g., Ephrins^66^).

Zinc-positive mossy fiber terminals and SPO-positive synapses are ectopically located in the dentate iml and oml. These aberrant synapses have been described in several models of epilepsy (e.g.^49,50^). This opens the question of whether Sema3E and PlexinD1 expression levels could be modulated in seizures as happens with other class III semaphorins^67–69^ and neuropilins^70–72^. In our experiments, we did not find a convulsive phenotype or epileptogenic activity in *Sema3E*^*0/0*^ mice. However, we found an increased excitability in the dentate gyrus revealed by the longer Up states and increased high-frequency components, mainly in the gamma (≈30–100 Hz) band. Interestingly, the dentate gyrus is the gateway to centripetal synaptic connections originating in the entorhinal cortex^3^. Moreover, neuronal activity of the entorhinal cortex has been demonstrated to modulate high-frequency gamma-like oscillatory activity at the level of the dentate gyrus^73^. Thus, it seems that the abnormal presence of ectopic mossy fibers and synaptic terminals found in the dentate gyrus of *Sema3E*^*0/0*^ mice might be related to the altered excitability present in these animals.

Furthermore, Sema3E and PlexinD1 expression levels could modulate synapse formation in the adult hippocampus. In this regard reduced levels of PlexinD1 decrease synapse density in neocortical neurons^74^ but increase dorsal thalamic input in striatal medium spiny neurons^75^. Our data suggesting an increase in zinc-positive sprouted fibers and boutons in the absence of *Sema3E* and *PlxnD1* might corroborate the results reported by Ding in striatum^75^. Although the mechanism of this participation remains unknown, we should consider that the effects of Sema3E/PlexinD1 are mediated in particular cell types by modulating β1-integrin function^76^ since mice lacking β1-integrin-mediated signalling displayed several abnormalities very similar to those reported in our study^77,78^. Thus, it is reasonable to consider that Sema3E/PlexinD1 signalling modulates cell adhesion and synapse formation for subsets of granule cells in adult hippocampus.

The alteration in the lamination of the granule cells seems likely to be associated with local dysregulation of cell proliferation in the subgranular zone and/or with radial migration deficits. In this regard, high levels of Sema3E/PlexinD1 signalling decrease the proliferation of malignant (e.g.^44,79,80^) and non-malignant cells (e.g.^81^). Thus, we may hypothesize that an absence of Sema3E might induce increased proliferation of subsets of stem cells in the subgranular layer. Indeed, since PlexinD1 is not expressed by DCX-positive cells, effects of Sema3E could circumscribe the regulation of GFAP-positive, type-1 radial glia-like hippocampal stem cells^82^. Further studies are needed to determine whether these effects are limited to adult stages or are acquired during postnatal stages.

In conclusion, our study describes new roles of Sema3E/PlexinD1 signalling during development and in adult hippocampal formation, and clearly expands current knowledge of the proposed functions.

## Material and Methods

### Mice

*Sema3E*^+*/0*^ mice and *PlxnD1*^*flox/flox*^ mice were reported in^38^ and^83^ respectively. Mice were maintained in heterozygous genotype and experiments were performed using embryos of the different genotypes. Animals were genotyped by PCR, as previously described elsewhere^38,83^. *Nestin-cre*^84^ and CD1 mice were purchased from Jackson Laboratories (Bar Harbor, ME, USA). *PlxnD1*-*eGFP* mice were obtained from the Mutant Mouse Regional Resource Center (MMRRC; University of California, CA, USA). Females were mated overnight and the mating day confirmed by the presence of a vaginal plug was considered as embryonic day 0.5 (E0.5). The day of birth, the night between E19.5 to E20.5, was considered as postnatal day 0 (P0). Animals were maintained in a pathogen-free barrier facility at the University of Barcelona animal facility. All experiments were performed under the guidelines and protocols of the Ethical Committee for Animal Experimentation (CEEA) of the University of Barcelona, and the protocol for the use of animals in this study was reviewed and approved by the CEEA of the University of Barcelona (CEEA approval #276/16 and 141/15).

### Cell transfection, explant and organotypic slice culture procedures

Embryonic CA1-3, entorhinal, subicular, ventrolateral and dorsal neocortical explants from E14.5 and E16.5 embryos were dissected out and placed in three-dimensional hydrogel of rat tail Collagen I^46^. Explants were cultured at 37 °C, 5% CO2 and 95% humidity in NeurobasalTM medium supplemented with B27 (Invitrogen) and glucose (6.5 mg/ml). After 2 days *in vitro* (DIV), genotypically identified cultures were paraformaldehyde-fixed and processed for βIII-tubulin (clone TUJ-1; Covance, BioLegend, San Diego, USA, Cat#MMS-435P) immunostaining (see^85^ for details). For cell confrontation assays, transfected COS1 cell aggregates expressing *SEAP* (control AP vector), *Sema3A-*AP, *Sema3F-*AP or *Sema3E-*AP were confronted with the neural explants in hydrogel matrices. Quantification of TUJ1-positive growing axons was performed as indicated^46^. Proximal/distal ratio was calculated (P/D < 1 chemorepulsion; P/D = 1 radial growth and P/D > 1 chemoattraction). *Sema3A*-AP, *Sema3E*-AP and *Sema3F*-AP plasmids were kindly provided by F. Mann. In addition, some experiments were conducted using commercial full-length cDNA clones *Sema3A* (MC205153), *Sema3E* (MC203244) and *Sema3F* (MC200862) purchased from OriGene Technologies (Rockville, MD, USA). As a second strategy, a growth cone collapse assay with entorhinal explants was also performed. Glass coverslips (12-mm Ø) were coated with Poly-L-Ornithine (0.01%, 1 h) and laminin (2 μg/ml, overnight). After washing, explants were placed in the same medium as above. After 3 DIV, their growth cones were visible and identifiable. To obtain the conditioned medium, COS1 cells were transfected with commercial *Sema3E, Sema3A, Sema3F* or *SEAP* expression vectors. Forty-eight hours after transfection, the medium was collected and 10 times concentrated in Millipore columns (Ultracel-30 membrane, Cat#UFC903024). Explants were incubated with 15% conditioned medium containing recombinant proteins or control media for 1 h. Cultures were then fixed in 4% paraformaldehyde and stained with Phalloidin-TRITC (Sigma-Aldrich, St. Louis, MO, USA). For quantification, a total of 50–100 growth cones were analysed for each condition and percentage of collapsed axons was measured. For organotypic slice preparation, the brains of newborn *Nestin-cre; PlxnD1*^*flox/*+^ (control, n = 5) and *Nestin-cre; PlxnD1*^*flox/flox*^ (mutant, n = 5) mice were dissected. Organotypic slices were prepared essentially as described^47^. After 7–10 days *in vitro*, the EH connection was traced with Byocitin^47^ and analysed.

### Axonal tracing

For 1,1′-dioctadecyl-3,3,3′3′-tetramethylindocarbocyanine perchlorate (DiI) tracing experiments, postnatal mice were fixed with 4% paraformaldehyde dissolved in 0.1 M phosphate buffer (pH 7.2–7.4). The brains were immersed in the same fixative solution at 4 °C. 300-μm thick horizontal sections were obtained using a Vibratome. Sections were injected with a crystal of DiI (Molecular Probes, Eugene, OR, USA) in the entorhinal cortex using a thin metal needle under microscope control. After injection, sections were stored in fixative solution at room temperature for 3 to 10 days in darkness. Thereafter, sections were counterstained with bisbenzimide (5 μM, Sigma-Aldrich), mounted in Mowiol^TM^ and photodocumented.

### Statistical processing

Statistical analysis of the obtained data (except electrophysiological studies) was performed using Bonferroni *post hoc* test (Multiple comparison test) using GraphPad Prism 6 (Mac OsX, Grahpad). Data are presented as mean ± standard error of the mean (S.E.M).

### *In Vivo* extracellular recordings

#### Surgical procedures

Four 12-month-old *Sema3E*^*0/0*^ mice and their respective littermates (*Sema3E*^+*/*+^) were used for extracellular recordings. Anesthesia was induced by the intraperitoneal administration of a mixture of ketamine (50 mg/kg) and medetomidine (1.3 mg/kg) and maintained, after tracheotomy, by the constant infusion of isoflurane (1%) in oxygen (100%). Atropine (0.3 mg/kg) and methylprednisolone (30 mg/kg) were administered subcutaneously to avoid respiratory secretions and prevent the appearance of edema. To study evoked epileptic discharges, kainic acid (10 mg/kg) was administered intraperitoneally. Body temperature was constantly monitored and kept at 37 °C by means of a thermal blanket (RWD Life Science, San Diego, CA, USA). Once stabilized, animals were placed on a stereotaxic frame (SR-6M, Narishige, London, UK) and a craniotomy was performed over the target area of the hippocampus (2.0 mm posterior to bregma, 1.0 mm lateral from midline^86^).

#### Extracellular recordings

Extracellular activity was recorded by means of 16-channel multielectrode probes (100 µm spacing, E16-100-S1-L6, Atlas Neuroengineering, Leuven, Belgium) previously marked with DiI for anatomical reconstruction, covering the DG and CA1 areas of the hippocampus and the overlying cerebral cortex. The signal was amplified (Multichannel systems (Germany)), digitized at 20 kHz and fed into a computer via a digitizer interface (CED 1401 interface and Spike2 software; Cambridge Electronic Design).

### Data analysis

Up and Down detection was done as previously described^87^. Briefly, the slow oscillation envelope, the envelope of the variance of the gamma-filtered local field potential (LFP) and the estimated multi-unit activity, were linearly combined to generate a time series where Up and Down states were singled out by setting a threshold. After detection, the mean duration of Up and Down states was calculated and Up state high-frequency components were analysed using Welch’s power spectrum density methods. Epileptic-like events were detected from the envelope of the variance of the raw LFP signal band-pass filtered between 3–6 Hz following previously described methods^88^. Data were analysed using the Wilcoxon rank-sum non-parametric statistical hypothesis testusing GraphPad Prism 6 (Mac OsX, Grahpad).

### Availability of materials and data

All data generated or analysed during this study are included in this published article (and its Supplementary information files).

## Electronic supplementary material

Supplementary information
