# Supplementary material for: New functions of Semaphorin 3E and its receptor PlexinD1 during developing and adult hippocampal formation
Source: Sci Rep. 2018 Jan 22;8:1381. doi: 10.1038/s41598-018-19794-0 (PMC5777998; doi:10.1038/s41598-018-19794-0)
Supplement: Supplementary file 1 — Supplementary information [file 41598_2018_19794_MOESM1_ESM.pdf]

Title: New functions of Semaphorin 3E and its receptor PlexinD1 during developing and adult hippocampal formation

Authors: Agata Mata[1,*], Vanessa Gil[1,*], Jeús Pérez-Clausell[2], Miguel Dasilva[3], Mari Carmen González-Calixto[5], Eduardo Soriano[6], José Manuel García-Verdugo[5], Maria V. Sanchez-Vives[3,4] and José Antonio del Río[1,¶].

* These authors contributed equally to this study

Addresses:

1)[1] Molecular and Cellular Neurobiotechnology, Institute for Bioengineering of Catalonia (IBEC), The Barcelona Institute of Science and Technology, Parc Científic de Barcelona, Barcelona, Spain. [2] Department of Cell Biology, Physiology and Immunology, Universitat de Barcelona, Barcelona, Spain. [3] Centro de Investigación Biomédica en Red sobre Enfermedades Neurodegenerativas (CIBERNED) and [4] Institut de Neurociències de la Universitat de Barcelona. Barcelona, Spain.

2) Department of Cell Biology, Physiology and Immunology, Universitat de Barcelona, Barcelona, Spain.

3) Systems Neuroscience, Institut d'Investigacions Biomèdiques August Pi i Sunyer (IDIBAPS), Barcelona, Spain.

4) ICREA, Barcelona, Spain.

5) Laboratory of Comparative Neurobiology, Institute Cavanilles, University of Valencia, CIBERNED, 46980 Valencia, Spain.

6)[1] Department of Cell Biology, Physiology and Immunology, Universitat de Barcelona, Barcelona, Spain. [2] Centro de Investigación Biomédica en Red sobre Enfermedades Neurodegenerativas (CIBERNED), Barcelona, Spain. [3] Vall d'Hebrón Institut de Recerca (VHIR), Barcelona, Spain. [4] ICREA, Barcelona, Spain and [5] Institut de Neurociències de la Universitat de Barcelona. Barcelona, Spain.

Supplementary information

Supplementary Figures 1-5

Supplementary Methods

References

Supplementary Figures

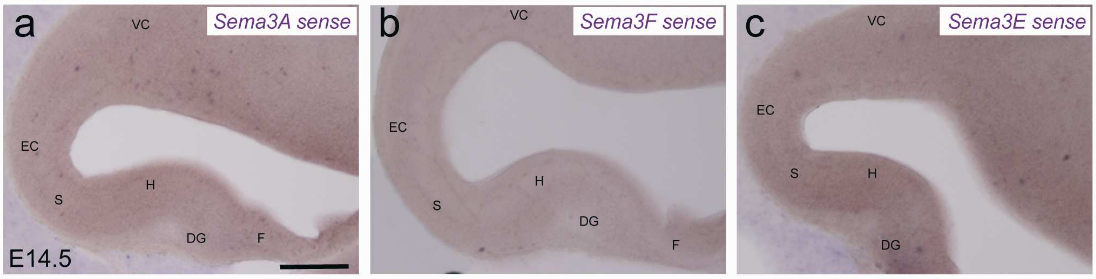

Supplementary Figure 1. Low-power photomicrographs illustrating the distribution of *Sema3A* (**a**), *Sema3F* (**b**) and *Sema3E* (**c**) sense transcripts in the hippocampal formation and adjacent ventrolateral cortex at E14.5. Abbreviations as in Figs. 1-6 and F = fimbria. Scale bars: **a** = 250 µm pertains to (**b-c**)

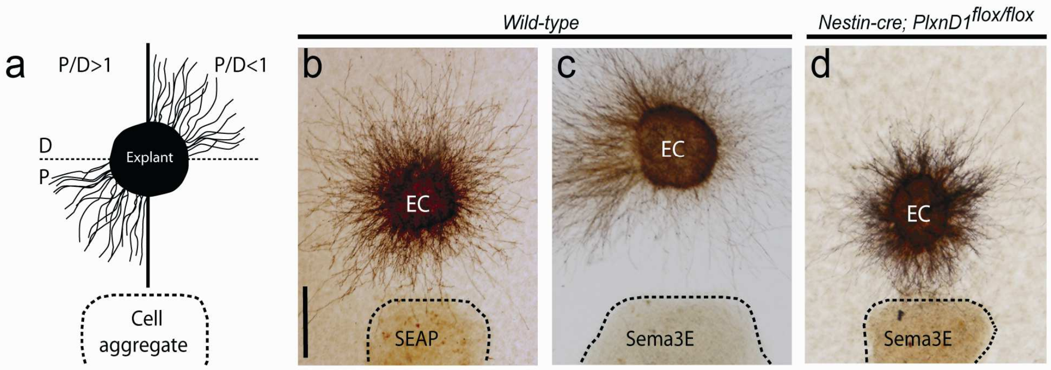

Supplementary Figure 2. (**a**) Schematic representation of axonal effect quantification in explants confrontation experiments. Dotted line delimits both the proximal (P) and the distal (D) quadrant in order to calculate the ratio P/D. Ratio P/D = 1 represents a radial pattern of growth; P/D < 1 indicates a chemorepulsive effect and P/D > 1 indicates a chemoattractive response. (**b-d)** Entorhinal explants obtained from wild-type **(b-c)** or PlexinD1-deficient (*Nestin-cre*; *PlxnD1*$^{flox/flox}$) mice **(d).** Note that the Sema3E-induced chemorepulsive effect observed in wild-type mice **(c)** is abolished in the absence of PlexinD1 receptor **(d).** Abbreviations: D = distal quadrant; P = proximal quadrant. Scale bars: **b**=250 µm pertains to **(c-d)**

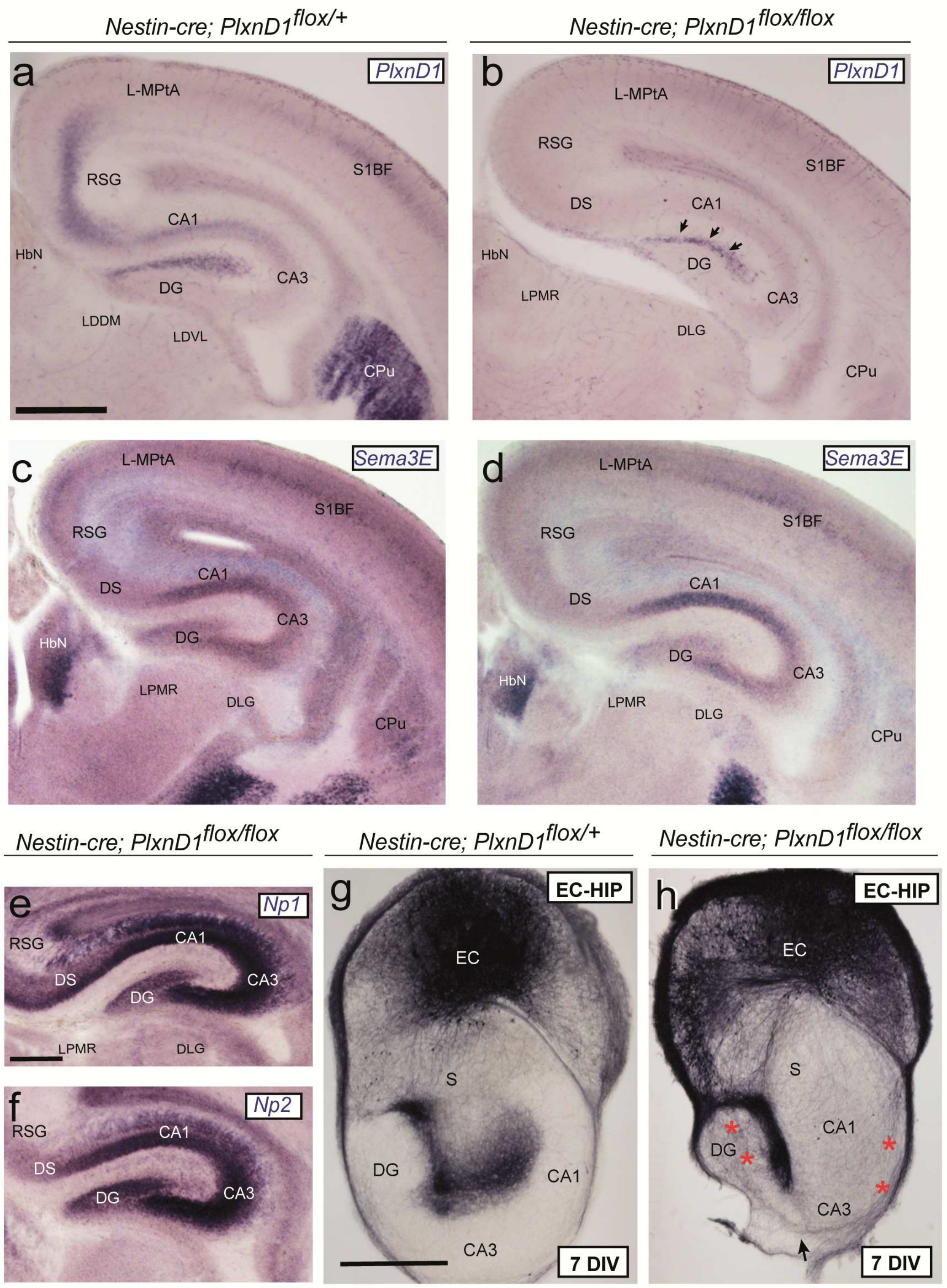

Supplementary Figure 3. (a-e) Low- and medium-power photomicrograhs illustrating *PlxnD1* (**a-b**), *Sema3E* (**c-d**), *Np1* (**e**) and *Np2* (**f**) mRNA distribution in control (**a, c,** *Nestin-cre*; *PlxnD1*$^{flox/+}$) and mutant (**b, d-f,** *Nestin-cre*; *PlxnD1*$^{flox/flox}$) mice at P0. Note the maintenance of the *PlxnD1* expression in early generated Cajal-Retzius cells in the hippocampus (arrows in **b**). No major changes were observed in

Sema3E (**d**) or *Np1-2* (**e-f**) mRNA distribution. (**g-h)** Low-power photomicrographs illustrating the traced EH connection in control (**g,** *Nestin-cre*; *PlxnD1*$^{flox/+}$) and mutant (**h**, *Nestin-cre*; *PlxnD1*$^{flox/flox}$*)* mice. Note the presence of ectopic fibers crossing CA1-3 (red asterisks and arrow in **h**) and the numerous ectopic fibers in the hilus (red asterisks in DG region) in mutant *Nestin-cre*; *PlxnD1*$^{flox/flox}$ EH slices. Abbreviations as in Fig. 1-6 and CPu = caudate putamen (striatum); DLG = dorsolateral geniculate nucleus; DS = dorsal subiculum; EC-HIP = entorhino-hippocampal co-culture; HbN = habenular nucleus; LDDM = laterodorsal thalamic nucleus, dorsomedial; LDVL = laterodorsal thalamic nucleus, ventrolateral; L-MPtA = lateral and medial parietal associational cortices; LPMR = lateroposterior thalamic nucleus, mediorostral; RSG = retrosplenial granular cortex and S1BF = first somatosensory barrel field. Scale bars: **a** = 150 μm pertains to (**b-d**); **e** = 100 μm pertains to **f**; **g** = 100 μm pertains to **h**

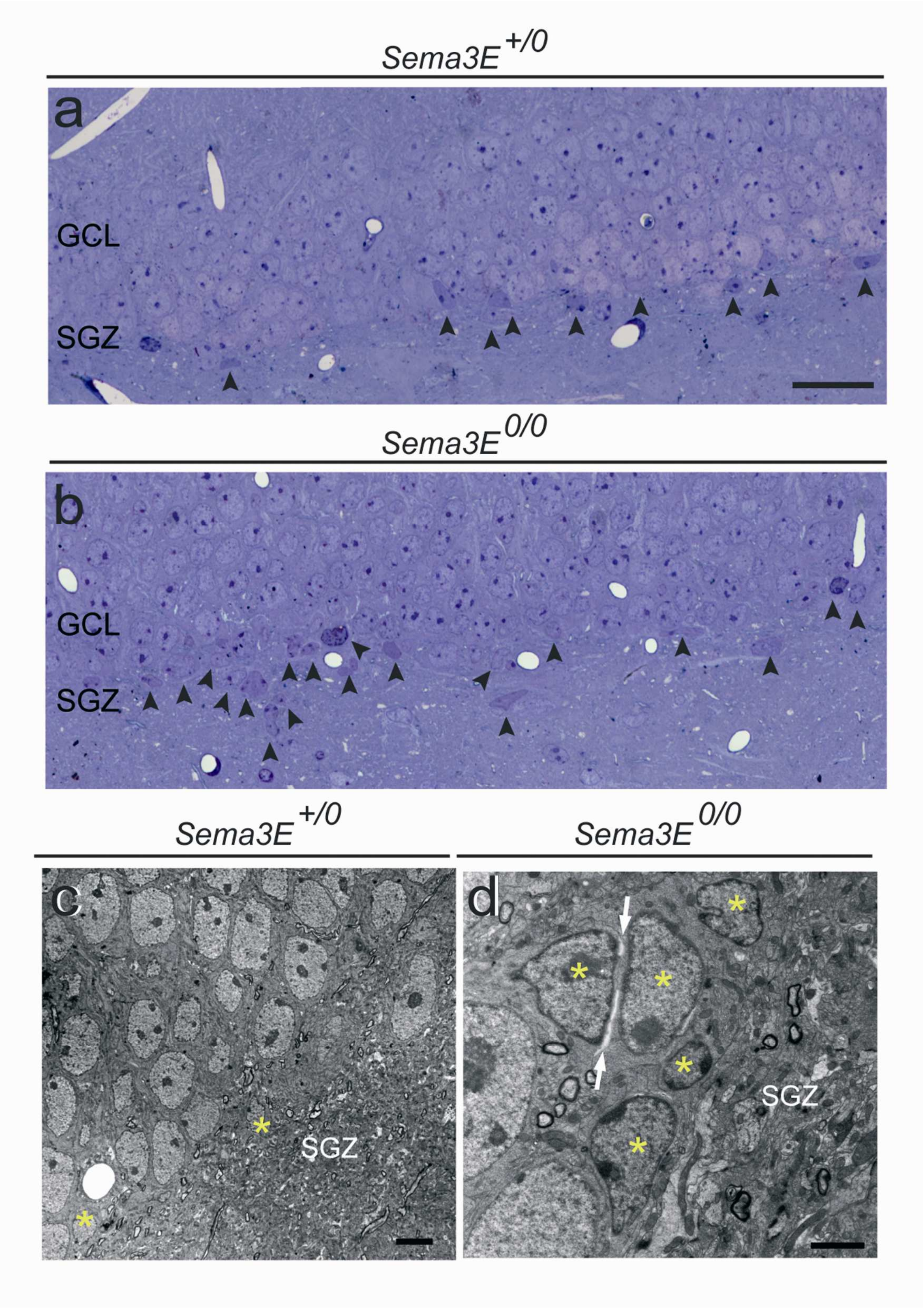

Supplementary Figure 4. Organization of the granule cell layer and the subgranular zone in *Sema3E*$^{0/0}$ mice**. (a-b)** Semithin (1.5-µm) sections of the upper pyramidal blade of the granule cell layer in *Sema3E*$^{+/0}$ (**a**) and *Sema3E*$^{0/0}$ (**b**) mice. Notice the presence of numerous clusters of immature cells in the subgranular zone (arrowheads) in the mutant mice compared to *Sema3E*$^{+/0}$. (**c-d)** High magnification

electron microscopy showing examples of inmature cells (asterisks) in stem cell niches of the subgranular zone in both genotypes. Notice the presence of different levels of chromatin condensation in these cells compared to postmitotic granule cells and extracellular empty spaces (arrows) typical of highly active cells during proliferation and migration in *Sema3E*$^{0/0}$ mice. Abbreviations as in Fig. 1-6. Scale bars: **a** = 25 µm pertains to **b**; **c=** 10µm; **d**= 2 µm

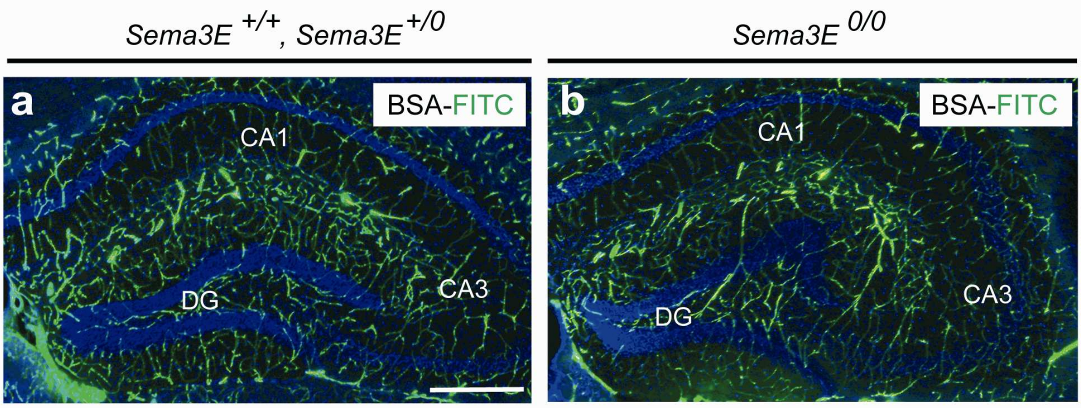

Supplementary Figure 5. (a-b) Low-power photomicrographs illustrating blood vessel distribution in the hippocampus proper and the dentate gyrus of control (*Sema3E*$^{+/+}$ *and Sema3E*$^{+/0}$) (**a**) and *Sema3E*$^{0/0}$ mice (**b**) after BSA-FITC staining. Abbreviations as in Figs. 1- 6. Scale bars: **a**=250 µm pertains to **(b)**

Supplementary Methods

***In situ* hybridization and immunohistochemistry**

In situ hybridization was carried out as described previously [1,2] on 40- or 80-µm thick frozen brain sections at several embryonic and postnatal stages (E14.5, E16.5, P0 and P2) of CD1 and P0 of *Nestin-cre;PlxnD1^{flox/flox} mice*. The following cDNA probes were used in this study: Sema3A (a gift from A. Chedotal), Sema3E and PlxnD1 (kindly provided by A. Kolodkin) and Sema3F, Np1 and Np2. Both sense and antisense riboprobes were labelled with digoxigenin following the manufacturer's instructions. For immunohistochemistry studies of frozen adult brain sections, tissue was incubated with primary antibodies overnight, followed by appropriate secondary antibodies. Tissue-binding primary antibody was detected using the Avidin-Biotin complex (ABC) method (Vector Labs). Briefly, free-floating sections were rinsed in 0.1 M PBS, and endogenous peroxidase activity was blocked by incubation in 3% H_2O_2 and 10% methanol dissolved in 0.1 M PBS. After extensive rinsing, sections were incubated in 0.1 M PBS containing 0.2% gelatin, 10% normal goat serum, 0.2% glycine and 0.2% Triton-X 100 for 1 hour at room temperature. Afterwards, sections were incubated for 24 hours at 4°C with the primary antibody: α-Calbindin and α-Calretinin, (Cat#CB38, 1:2000 and Cat#7697, 1:500 diluted respectively, Swant Antibodies, Marly, Switzerland), Doublecortin (DCX, 1:500, Santa Cruz Biotechnology, USA, Cat#SC-8066), GFAP (1:1500, Dako, Glostrup, Denmark, Cat#Z0334), GFP (1:500, Invitrogen, Thermo Fisher Biotechnologies, Cat#A11122), NeuN (1:1000, Millipore, Merck, Darmstadt, Germany, Cat#MAB377), Parvalbumin (PARV, 1:2000, Swant Antibodies,Cat#PV28), Prox-1 (1:2000, Millipore, Cat#AB5475), Synaptoporin (SPO, 1:200, Synaptic systems, Gottingen, Germany, Cat#102002). After that, sections were incubated with secondary biotinylated antibodies (2 hours, 1:200 diluted) and streptavidin-horseradish peroxidase complex (2 hours, 1:400 diluted). Peroxidase activity was developed with 0.025% diaminobenzidine (DAB) and 0.003% hydrogen peroxide. After rinsing, sections were mounted onto slides, dehydrated, and coverslipped with Eukitt™ (Merck). In parallel, additional sections were processed for double immunofluorescences using Alexa Fluor 488- and Alexa Fluor 568-tagged secondary antibodies (Molecular Probes, Eugene, USA). For determination of blood vessel distribution in the hippocampus, anaesthetized mice received a single injection of BSA-FITC (Sigma-Aldrich, Cat#A9771, 25 mg/ml in 0.1M PBS) [3] in the right ventricle. After 5 minutes, mice were killed and their brains immersion-fixed in 4% buffered paraformaldehyde overnight. 60-µm thick Vibratome (Leica) sections were obtained, counterstained with Bisbenzimide and mounted in Mowiol™. All samples were photodocumented using an Olympus BX61 microscope equipped with a cooled digital DP72L camera and

figures were created using Adobe Illustrator CS6 software (Adobe systems). In addition, *Sema3E*$^{+/0}$ (n=4) and *Sema3E*$^{0/0}$ (n=4) mice were processed for transmission electron microscopy as previously described [4].

5´-Bromo-2-deoxyuridine (BrdU) labelling

Adult mice (2-3 month old, n = 4 *Sema3E*$^{0/0}$, n= 4 *Sema3E*$^{+/0}$ and n= 4 *Sema3E*$^{+/+}$ from two different litters) were intraperitoneally injected with BrdU (50 mg/kg b.w., one pulse per day) for 4 days [5]. One week later, mice were processed for BrdU immunostaining as described [5,6,7]. After photodocumentation, the BrdU-positive cells located in the dentate gyrus and the outermost portion of the granule cell layer of the dentate gyrus were counted.

Selenite-silver staining

The selenite-silver staining for zinc-rich projections (i.e., glutamatergic mossy fibers) was performed essentially as previously described [8]. Adult mice were injected intraperitoneally with Na_2SeO_3 at a final dose of 20 mg/kg. After 1 h, animals were anesthetized (chloral hydrate, 700 mg/kg, intraperitoneally) and brains were quickly removed and frozen in carbonic ice. Four series of 30 µm-thick cryostat sections were cut and stored at -20°C until staining. Sections were developed in the dark for 60-80 minutes in a silver-amplifying solution: 60 mL 50% (w/v) gum arabic, 10 mL 2 M sodium citrate buffer, 10 mL 8.5% (w/v) hydroquinone, 10 mL 1.2% (w/v) silver lactate and 10 mL distilled water. Sections were then thoroughly rinsed in distilled water and in 5% sodium thiosulphate for 12 min, dehydrated in ethanol and coverslipped with Eukitt™.

References

1. Mingorance, A., X. Fontana, et al. (2004). "Regulation of Nogo and Nogo receptor during the development of the entorhino-hippocampal pathway and after adult hippocampal lesions." Mol Cell Neurosci **26**(1): 34-49

2. Tomas-Roig, J., F. Piscitelli, et al. (2016). "Social defeat leads to changes in the endocannabinoid system: An overexpression of calreticulin and motor impairment in mice." Behav Brain Res **303**: 34-43.

3. Giannoni, P., M. Arango-Lievano, et al. (2016). "Cerebrovascular pathology during the progression of experimental Alzheimer's disease." Neurobiol Dis **88**: 107-117.

4. Seri, B., J. M. Garcia-Verdugo, et al. (2001). "Astrocytes give rise to new neurons in the adult mammalian hippocampus." J Neurosci **21**(18): 7153-7160.

5. Fontana, X., J. Nacher, et al. (2006). "Cell proliferation in the adult hippocampal formation of rodents and its modulation by entorhinal and fimbria-fornix afferents." Cereb Cortex **16**(3): 301-312.

6. del Rio, J. A. and E. Soriano (1989). "Immunocytochemical detection of 5'-bromodeoxyuridine incorporation in the central nervous system of the mouse." Brain Res Dev Brain Res **49**(2): 311-317.

7. Soriano, E. and J. A. Del Rio (1991). "Simultaneous immunocytochemical visualization of bromodeoxyuridine and neural tissue antigens." J Histochem Cytochem **39**(3): 255-263.

8. Danscher, G. (1982). "Exogenous selenium in the brain. A histochemical technique for light and electron microscopical localization of catalytic selenium bonds." Histochemistry **76**(3): 281-293.
